# NHR‐49/HNF4 integrates regulation of fatty acid metabolism with a protective transcriptional response to oxidative stress and fasting

**DOI:** 10.1111/acel.12743

**Published:** 2018-03-05

**Authors:** Grace Y. S. Goh, Johnathan J. Winter, Forum Bhanshali, Kelsie R. S. Doering, Regina Lai, Kayoung Lee, Elizabeth A. Veal, Stefan Taubert

**Affiliations:** ^1^ Graduate Program in Cell & Developmental Biology University of British Columbia Vancouver BC Canada; ^2^ Centre for Molecular Medicine and Therapeutics Vancouver BC Canada; ^3^ BC Children's Hospital Research Institute Vancouver BC Canada; ^4^ Institute for Cell and Molecular Biosciences Newcastle University Newcastle upon Tyne UK; ^5^ Newcastle University Institute for Ageing Newcastle University Newcastle upon Tyne UK; ^6^ Department of Medical Genetics University of British Columbia Vancouver BC Canada

**Keywords:** fasting, FMO, HNF4, oxidative stress, PPAR, ROS

## Abstract

Endogenous and exogenous stresses elicit transcriptional responses that limit damage and promote cell/organismal survival. Like its mammalian counterparts, hepatocyte nuclear factor 4 (HNF4) and peroxisome proliferator‐activated receptor α (PPARα), *Caenorhabditis elegans* NHR‐49 is a well‐established regulator of lipid metabolism. Here, we reveal that NHR‐49 is essential to activate a transcriptional response common to organic peroxide and fasting, which includes the pro‐longevity gene *fmo‐2/flavin‐containing monooxygenase*. These NHR‐49‐dependent, stress‐responsive genes are also upregulated in long‐lived *glp‐1/notch receptor* mutants, with two of them making critical contributions to the oxidative stress resistance of wild‐type and long‐lived *glp‐1* mutants worms. Similar to its role in lipid metabolism, NHR‐49 requires the mediator subunit *mdt‐15* to promote stress‐induced gene expression. However, NHR‐49 acts independently from the transcription factor *hlh‐30/TFEB* that also promotes *fmo‐2* expression. We show that activation of the p38 MAPK, PMK‐1, which is important for adaptation to a variety of stresses, is also important for peroxide‐induced expression of a subset of NHR‐49‐dependent genes that includes *fmo‐2*. However, organic peroxide increases NHR‐49 protein levels, by a posttranscriptional mechanism that does not require PMK‐1 activation. Together, these findings establish a new role for the HNF4/PPARα‐related NHR‐49 as a stress‐activated regulator of cytoprotective gene expression.

## 1 INTRODUCTION

The ability to respond to acute stress conditions is vital to prevent or limit organismal damage. For instance, transcriptional responses promote adaptation and survival under stress conditions. Impaired stress responses contribute to human age‐related diseases such as diabetes and neurodegenerative disorders (Hetz, Chevet & Harding, 2013; Lin & Beal, 2006), and likely also contribute to aging (Hekimi, Lapointe & Wen, 2011; Shore & Ruvkun, 2013). However, these adaptive responses also protect pathogens and cancer cells against cytotoxic drugs and the immune system (Leprivier, Rotblat, Khan, Jan & Sorensen, 2015; Rankin & Giaccia, 2016). Accordingly, there is widespread interest in defining the mechanisms involved in coordinating these transcriptional responses.

Transcription factors of the nuclear factor (erythroid‐derived 2)‐like 2 (Nrf2) family are required to activate many cytoprotective genes in animals. In the nematode *Caenorhabditis elegans*, the Nrf2 ortholog SKiNhead (SKN‐1) implements a wide range of homeostatic and stress responses in response to internal and external stimuli (Blackwell, Steinbaugh, Hourihan, Ewald & Isik, 2015). Although the transcription factors DAF‐16, HSF‐1, HLH‐30, and HIF‐1 activate a variety of stress/pathogen‐protective responses, SKN‐1 is especially important to maintain redox balance and defend against oxidative stress caused by xenobiotics that target GSH and cause protein unfolding (Blackwell et al., 2015; Miranda‐Vizuete & Veal, 2016). Nevertheless, distinct responses are initiated in response to different oxidants (Blackwell et al., 2015; Wu, Deonarine, Przybysz, Strange & Choe, 2016). For example, the organic peroxide tert‐butyl‐hydroperoxide (tBOOH) elicits a transcriptional response that is largely *skn‐1*‐independent (Oliveira et al., 2009). Notably, *mdt‐15*, a subunit of the mediator transcriptional coregulator that is required for *skn‐1*‐dependent stress responses, is also required for the *skn‐1‐*independent transcriptional response to tBOOH (Goh et al., 2014; Taubert, Hansen, Van Gilst, Cooper & Yamamoto, 2008). One of the most highly tBOOH‐induced, *mdt‐15*‐dependent, *skn‐1*‐independent genes is the flavin‐containing monooxygenase *fmo‐2*, whose HIF‐1‐dependent induction in response to hypoxia and dietary restriction (DR) is important for the associated increase in lifespan. Indeed, *fmo‐2* is induced in several *C. elegans* longevity paradigms (Bennett et al., 2017; Leiser et al., 2015). Elucidating how *fmo‐2* and other genes induced under these conditions are regulated is important to understand how these responses become defective during aging or in disease (Hekimi et al., 2011; Hetz et al., 2013; Lin & Beal, 2006; Shore & Ruvkun, 2013).

Here, we have used *fmo‐2* as a marker to investigate SKN‐1‐independent stress response mechanisms by determining how this pro‐longevity gene is activated in response to organic peroxide and fasting. We found that the nuclear hormone receptor (NHR)‐49, an HNF4/PPARα ortholog and established regulator of *C. elegans* lipid metabolism and longevity (Pathare, Lin, Bornfeldt, Taubert & Van Gilst, 2012; Ratnappan et al., 2014; Van Gilst, Hadjivassiliou, Jolly & Yamamoto, 2005; Van Gilst, Hadjivassiliou & Yamamoto, 2005), is also activated by and required for oxidative stress resistance. We show that NHR‐49 and MDT‐15 are both required for the increased expression of *fmo‐2* as part of a stress response program activated by organic peroxide, fasting, and in long‐lived, germline‐less *glp‐1* mutants. Together, our data suggest that the function of NHR‐49 is not confined to regulating lipid metabolism, but also involves activation of a stress‐protective transcriptional program.

## 2 RESULTS

### 2.1 *nhr‐49* is required to induce an oxidative stress and fasting response program

**Figure 1:**
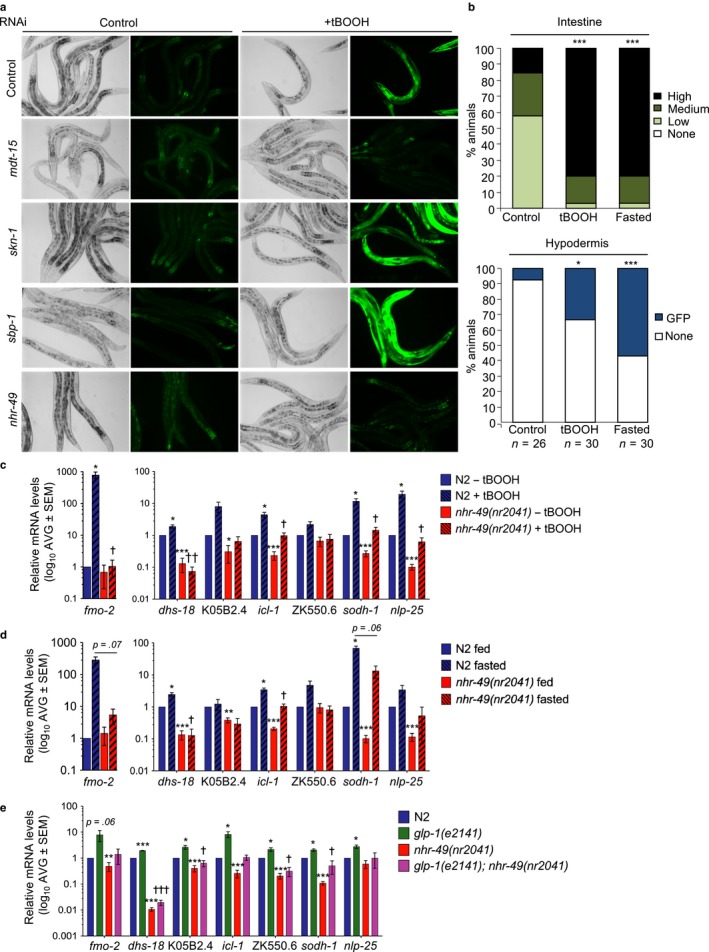
*nhr‐49* is required for a stress response program activated by organic peroxide, fasting, and *glp‐1* mutation. (a) Micrographs show *fmo‐2p::gfp* worms grown on control, *mdt‐15*,* skn‐1*,* sbp‐1*, and *nhr‐49* RNAi after 3 hr on 10 mm tBOOH. See also Tables S1 and S2 and Figure S1. (b) Quantification of GFP levels in *fmo‐2p::gfp* worms in the intestine and hypodermis after 3 hr on 10 mm tBOOH or 12 hr of fasting. (c) Fold changes of mRNA levels (relative to untreated wild‐type N2) in L4 N2 and *nhr‐49*(*nr2041*) worms treated with 7.5 mm tBOOH for 4 hr (*n* = 5). ^*,**,***^ *p *<* *.05, .01, or .001 vs. N2 untreated worms. ^†,††^ *p *<* *.05 or .01 vs. N2‐treated worms (unpaired Student's *t* test corrected for multiple comparisons using the Holm–Sidak method). (d) Same as (c), but with 8 hr of fasting (*n* = 3). (e) Fold changes of mRNA levels (relative to N2 wild‐type) in L4 N2, *glp‐1*(*e2141*), *nhr‐49*(*nr2041*), and *nhr‐49*(*nr2041*)*; glp‐1*(*e2141*) worms. ^*,**,***^ *p *<* *.05, .01, or .001 vs. N2 worms. ^†,†††^ *p *<* *.05 or .001 vs. *glp‐1*(*e2141*) worms

To screen for factors cooperating with MDT‐15 in the tBOOH‐induced expression of *fmo‐2*, we generated a transgenic strain expressing a transcriptional *fmo‐2p::gfp* reporter (Figure S1a). This reporter was constitutively expressed in some neurons and weakly active in the intestine of well‐fed animals, but strongly induced in the intestine, hypodermis, and pharynx following exposure to tBOOH (Figure 1a,b). The *fmo‐2p::gfp* reporter was also activated by DTT and H_2_O_2_, but not by arsenite and cadmium (Figure S1b). Thus, consistent with other studies, our transcriptional reporter was strongly activated by tBOOH but not by stimuli that activate SKN‐1‐dependent gene expression (Goh et al., 2014; Oliveira et al., 2009).

To identify genes required for tBOOH‐induced expression of *fmo‐2p::gfp*, we used RNA interference (RNAi) to deplete 25 candidate transcription factors that bind either MDT‐15 or the *fmo‐2* promoter (Tables S1 and S2). As expected, *mdt‐15* RNAi completely abrogated tBOOH‐induced *fmo‐2* expression, whereas neither *skn‐1/Nrf* nor *sbp‐1/SREBP* were required (Figure 1a; Tables S1). Of the tested candidates, *nhr‐49* was the only RNAi clone that consistently blocked *fmo‐2* induction by tBOOH (Figure 1a; Tables S1 and S2).

*nhr‐49* encodes an NHR that binds MDT‐15 and is required for tBOOH resistance (Goh et al., 2014; Taubert, Van Gilst, Hansen & Yamamoto, 2006). NHR‐49 and MDT‐15 regulate lipid metabolism and are needed to induce several lipid metabolism genes in fasted worms (Taubert et al., 2006; Van Gilst et al., 2005; Van Gilst, Hadjivassiliou & Yamamoto, 2005). As *fmo‐2* is also induced by fasting (Leiser et al., 2015), we tested whether *nhr‐49* is required for *fmo‐2* induction in fasted worms. Real‐time quantitative PCR (qPCR) analysis showed that *fmo‐2* mRNA levels are strongly induced by tBOOH and by fasting, and this induction was blocked in *nhr‐49*(*nr2041*) null mutants (Figure 1c,d).

**Figure 2:**
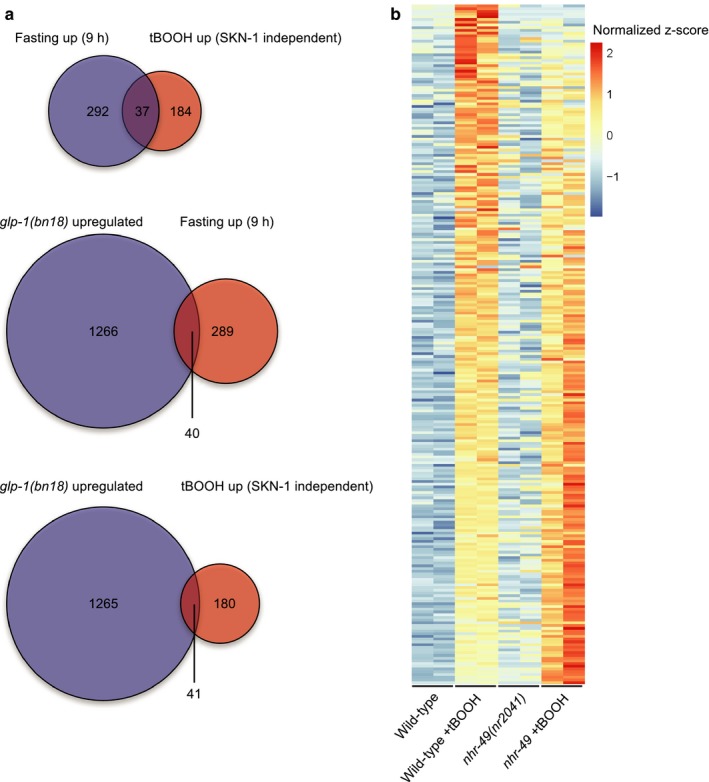
*nhr‐49* is required for tBOOH response. (a) Venn diagrams show the overlaps between genes upregulated by tBOOH in a SKN‐1‐independent manner (Oliveira et al., 2009), upregulated after 9 hr of fasting (Uno et al., 2013), and upregulated in the *glp‐1*(*bn18*) mutant (Steinbaugh et al., 2015). *p *<* *2.2 × 10^−16^, *p *=* *.0001892, and *p *=* *1.598 × 10^−09^, respectively (Fisher's exact test). (b) The heat map shows the z‐score transformed expression values of the 250 genes that are induced more than fourfold and with FDR <0.05 in wild‐type worms by tBOOH. These genes are arranged from highest to lowest difference in z‐score in wild‐type +tBOOH vs. *nhr‐49 *+* *tBOOH. Genes are colored based on their z‐score normalized to the average across all four conditions (untreated wild‐type worms, tBOOH‐treated wild‐type worms, untreated *nhr‐49*(*nr2041*) mutants, and tBOOH‐treated *nhr‐49*(*nr2041*) mutant). Note, for each condition, two biological replicates are shown. See also Tables [Link], [Link], [Link]

The discovery that *nhr‐49* was required to induce *fmo‐2* in response to either tBOOH or fasting raised the possibility that these stresses activate a common biological response. Indeed, meta‐analysis revealed a significant overlap between genes upregulated by tBOOH and by fasting (Figure 2a), with qPCR analysis confirming that 20 of these genes are upregulated in response to either stress (Figure 1c,d, data not shown). Notably, the tBOOH‐ and fasting‐induced expression of seven of these genes was impaired in *nhr‐49*(*nr2041*) worms, indicating that NHR‐49 is important for the shared transcriptional response to these stresses (Figure 1c,d). *nhr‐49* is also required for metabolic remodeling and increased lifespan due to loss of *glp‐1/notch receptor* activity in a *glp‐1*(*e2141*) mutant (Ratnappan et al., 2014). Notably, meta‐analysis of transcriptome data revealed significant overlaps between genes induced in *glp‐1*(*bn18*) mutants (Steinbaugh et al., 2015) and induced by either tBOOH or fasting (Figure 2a). Notably, qPCR analysis showed that the expression of *fmo‐2* and other tBOOH‐ and fasting‐induced genes was also increased in long‐lived *glp‐1*(*e2141*) mutants and that this was abrogated by loss of NHR‐49 (Figure 1e).

We also used RNA‐seq to compare the transcriptomes of wild‐type and *nhr‐49*(*nr2041*) worms before and following exposure to tBOOH (Figure 2b;Tables S3 and S4). We found that tBOOH induced 250 genes more than fourfold in wild‐type. Consistent with our meta‐analysis with published datasets (Figure 2a), there was a significant overlap between these 250 tBOOH‐responsive genes and previously published tBOOH, fasting, and *glp‐1*‐regulated genes (Table S5). Notably, of the 250 genes, the induction of 75 was compromised by loss of *nhr‐49*, including *fmo‐2* and *nlp‐25* (FDR > 0.05 and >twofold reduced induction in *nhr‐49* mutants vs. wild‐type); in contrast, these genes were not significantly (FDR < 0.05) deregulated by loss of *nhr‐49* in the absence of tBOOH (Table S4). Together, these data suggest that *nhr‐49* is required for increased expression of a shared set of genes following oxidative stress, fasting, and in at least one long‐lived mutant.

### 2.2 Active *nhr‐49* and its coactivator *mdt‐15* are sufficient for increased expression of stress‐responsive genes

NHR‐49 dimerizes with NHR‐13, NHR‐66, and NHR‐80 to regulate genes involved in lipid metabolism (Folick et al., 2015; Pathare et al., 2012). However, the tBOOH‐induced expression of *fmo‐2*, K05B2.4, and *icl‐1* was largely unchanged in *nhr‐13*(*ok796*), *nhr‐66*(*ok940*), and *nhr‐80*(*tm1011*) null mutants (Figure S2a). This suggests that NHR‐49 does not partner with these NHRs in the tBOOH response.

**Figure 3:**
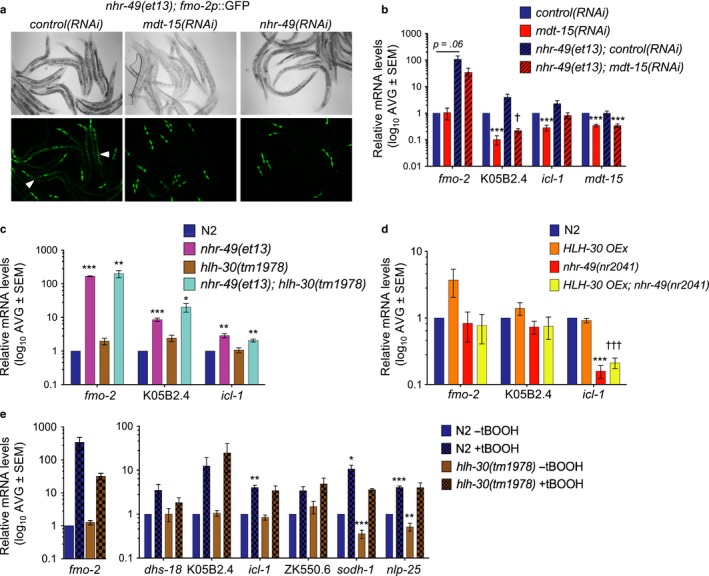
Stress response gene induction by *nhr‐49* requires *mdt‐15* but not *hlh‐30*. (a) Micrographs of *nhr‐49*(*et13*)*; fmo‐2p::gfp* worms grown on control, *mdt‐15*, and *nhr‐49* RNAi. Arrowheads indicate intestinal GFP. (b) Fold changes of mRNA levels (relative to N2 *control*(*RNAi*) worms) in L4 N2 and *nhr‐49*(*et13*) worms treated with control and *mdt‐15* RNAi (*n* = 5). ^***^ *p *<* *.001 vs. N2 *control*(*RNAi*) worms, ^†^ *p *<* *.05 vs. *nhr‐49*(*et13*) *control*(*RNAi*) worms (unpaired Student's *t* test corrected for multiple comparisons using the Holm–Sidak method). (c) Fold changes of mRNA levels (relative to wild‐type N2 worms) in L4 N2, *nhr‐49*(*et13*), *hlh‐30*(*tm1978*), and *hlh‐30*(*tm1978*); *nhr‐49*(*et13*) worms (*n* ≥ 3). ^*,**,***^ *p *<* *.05, .01, or .001 vs. N2 worms (unpaired Student's *t* test corrected for multiple comparisons using the Holm–Sidak method). (d) Fold changes of mRNA levels (relative to wild‐type N2 worms) in L4 N2 worms, N2 worms overexpressing *hlh‐30p::hlh‐30::gfp*,* nhr‐49*(*nr2041*) mutants, and *nhr‐49*(*nr2041*) mutants overexpressing *hlh‐30p::hlh‐30::gfp* (*n* ≥ 3). ^***^ *p *<* *.001 vs. N2 worms, ^†††^ *p *<* *.05 vs. N2 worms overexpressing *hlh‐30p::hlh‐30::gfp* (unpaired Student's *t* test corrected for multiple comparisons using the Holm‐Sidak method). (e) Fold changes of mRNA levels (relative to wild‐type N2‐untreated worms) in L4 N2 and *hlh‐30*(*tm1978*) worms treated with 7.5 mm tBOOH for 4 hr (*n* = 5). Statistics as in (b). ^**,***^ *p *<* *.01, or .001 vs. N2 untreated worms. See also Figure S3

To test whether increased NHR‐49 activity alone is sufficient to activate these stress‐responsive genes, we crossed the *fmo‐2p::gfp* reporter into the *nhr‐49*(*et13*) gain‐of‐function (gof) mutant (Lee, Goh, Wong, Klassen & Taubert, 2016). We observed constitutive intestinal and pharyngeal fluorescence in the *fmo‐2p::gfp; nhr‐49*(*et13*) strain, which was ablated by *nhr‐49* RNAi, suggesting that increased NHR‐49 activity is sufficient to activate *fmo‐2* in these tissues (Figure 3a). qPCR analysis of *nhr‐49*(*et13*) worms also revealed higher levels of *fmo‐2* and K05B2.4 mRNA, although other tested tBOOH‐ and fasting‐induced genes were only modestly induced (Figure 3b, data not shown). In contrast, the expression of arsenite‐responsive genes (*gst‐4*,* gcs‐1*,* gst‐6*,* ptps‐1*), which require *skn‐1* and *mdt‐15* for activation (Goh et al., 2014; Oliveira et al., 2009), was not increased in *nhr‐49* gof mutants (Figure S2b). Thus, our data suggest that NHR‐49‐dependent oxidative stress gene activation is distinct from the SKN‐1‐dependent response to arsenite.

*mdt‐15* is an essential coactivator for SKN‐1 in the arsenite response and for NHR‐49 in the regulation of lipid metabolism genes (Goh et al., 2014; Taubert et al., 2006). Hence, we tested whether MDT‐15's role as an NHR‐49 coactivator might underlie its role in tBOOH‐induced, SKN‐1‐independent gene expression. Indeed, *mdt‐15* RNAi abolished the intestinal fluorescence in *fmo‐2p::gfp; nhr‐49*(*et13*) worms and the increased K05B2.4 mRNA levels in *nhr‐49*(*et13*) worms (Figure 3a,b). Elevated pharyngeal expression of *fmo‐2p::gfp* was unaffected by either *mdt‐15* or *nhr‐49* RNAi, likely reflecting RNAi resistance of this tissue and possibly explaining the limited effect of *mdt‐15* RNAi on whole‐worm *fmo‐2* mRNA levels (Figure 3b). Altogether, these data suggest that, in the presence of MDT‐15, active NHR‐49 is sufficient to induce *fmo‐2* and K05B2.4, consistent with MDT‐15 acting as a coactivator for NHR‐49‐driven, tBOOH‐induced gene expression.

### 2.3 *nhr‐49* acts independently of *hlh‐30* to activate *fmo‐2* and other stress‐responsive genes

The intestinal induction of *fmo‐*2 by fasting or hypoxia requires the transcription factors *hlh‐30/TFEB* and *hif‐1/HIF* (Leiser et al., 2015), master regulators of *C. elegans* autophagy, and hypoxia gene programs, respectively (Lapierre et al., 2013; O'Rourke & Ruvkun, 2013; Powell‐Coffman, 2010). Moreover, *hlh‐30* is required for the long lifespan of *glp‐1*(*e2141*) mutants (Nakamura et al., 2016). *hif‐1* RNAi did not prevent tBOOH‐induced expression of *fmo‐2p::gfp* (data not shown). However, reminiscent of its activation following fasting (O'Rourke & Ruvkun, 2013), we observed a transient increase in nuclear levels of a HLH‐30::GFP fusion protein in response to tBOOH (Figure S3a). Thus, we examined whether *hlh‐30* and *nhr‐49* might function in the same stress response pathway. However, qPCR analysis revealed that three genes were induced similarly in the *nhr‐49*(*et13*) single and the *hlh‐30*(*tm1978*)*; nhr‐49*(*et13*) double mutant, suggesting that *hlh‐30* is dispensable for the activation of these genes by NHR‐49 (Figure 3c). Moreover*,* these genes were not significantly upregulated by transgenically overexpressing *hlh‐30*, either in the presence or in the absence of NHR‐49 (Figure 3d). Lastly, *hlh‐30*(*tm1978*) mutation only weakly reduced *fmo‐2* induction by tBOOH (statistically not significant, Figures 3e and S3b–d). In contrast, *hlh‐30* was partially required for *fmo‐2* induction by fasting, as reported (Leiser et al., 2015), and for *sodh‐1* expression, under both stress and non‐stress conditions (Figures 3e and S3b–d). These data suggest that NHR‐49 and HLH‐30 act in parallel, or that NHR‐49 acts downstream of HLH‐30, to promote the transcriptional response to organic peroxide. Indeed, our inability to obtain an *nhr‐49*(*nr2041*)*; hlh‐30*(*tm1978*) double mutant (see Experimental Procedures) suggests that these genes act nonredundantly in parallel pathways.

### 2.4 NHR‐49 levels increase following oxidative stress

**Figure 4:**
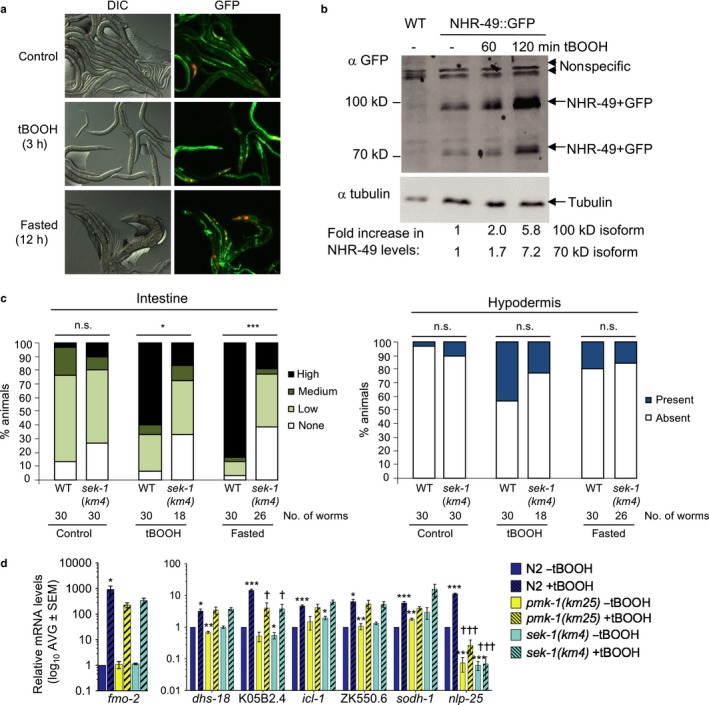
Regulation of NHR‐49 protein levels by tBOOH is *sek‐1*‐dependent. (a) Micrographs show *Pnhr‐49::nhr‐49::gfp* worms in normal and stress conditions. (b) Immunoblots on lysates of wild‐type (WT) and *Pnhr‐49::nhr‐49::gfp* (NHR‐49::GFP) worms, untreated or treated with 10 mm tBOOH. Two bands (~100 and ~70 kDa) are detected in worms expressing NHR‐49::GFP fusion proteins; arrowhead indicates nonspecific bands also detected in WT worms. Tubulin = loading control. Fold increases in NHR‐49::GFP levels (normalized to tubulin), relative to the levels of each isoform in untreated animals, are indicated. (c) Quantification of intestinal and hypodermal GFP intensity in *fmo‐2p::gfp* and *sek‐1*(*km4*)*; fmo‐2p::gfp* worms following exposure to tBOOH and fasting. (d) Fold changes of mRNA levels (relative to untreated N2 wild‐type) in L4 N2, *pmk‐1*(*km25*), and *sek‐1*(*km4*) worms treated with 7.5 mm tBOOH for 4 hr (*n* = 4). ^*,**,***^ *p *<* *.05, .01, or .001 vs. N2 untreated worms. ^†,†††^ *p *<* *.05 or .001 vs. wild‐type treated worms (unpaired Student's *t* test corrected for multiple comparisons using the Holm–Sidak method). See also Figure S4

Next, we examined whether NHR‐49 is activated by oxidative stress. NHR‐49 levels are increased in *glp‐1* mutants due to DAF‐16‐ and TCER‐1‐driven increases in *nhr‐49* mRNA levels (Ratnappan et al., 2014). Hence, we used an *Pnhr‐49::nhr‐49::GFP* transgene that encodes all known *nhr‐49* isoforms (Figure S4a; Ratnappan et al., 2014) to test whether levels or localization of NHR‐49::GFP fusion proteins also change in response to tBOOH or fasting. The high basal levels of nuclear NHR‐49::GFP made stress‐induced changes difficult to detect by microscopy (Figure 4a). However, Western blot analysis of whole‐worm lysates showed that bands with mobilities of ~100 kDa and ~70 kDa, absent from animals lacking the transgene, were reduced by *nhr‐49* RNAi and increased upon exposure to tBOOH (Figures 4b and S4c). Notably, *nhr‐49* mRNA levels were not induced by this exposure to tBOOH (Figure S4d), suggesting that NHR‐49 levels are posttranscriptionally raised in response to tBOOH. Intriguingly, further electrophoretic separation of proteins revealed the presence of a less mobile ~100kD NHR‐49::GFP isoform following tBOOH treatment (Figures 4b and S4c). This could reflect specific tBOOH‐induced increased levels of one of the larger NHR‐49::GFP isoforms (Figure S4a), but is also consistent with the predominant isoform(s) becoming posttranslationally modified, for example, by phosphorylation.

### 2.5 Stress activation of the p38 MAPK, PMK‐1, is required for the tBOOH‐induced expression of some NHR‐49‐dependent genes but dispensable for increase in NHR‐49 protein levels

The conserved p38 mitogen‐activated protein kinase (MAPK) PMK‐1 is activated by phosphorylation by the SEK‐1 MAPKK in response to many stresses, including tBOOH. PMK‐1‐dependent phosphorylation of SKN‐1 is vital for stress‐induced increases in its activity (Blackwell et al., 2015; Inoue et al., 2005). Hence, we hypothesized that these kinases might be required for tBOOH‐induced increases in NHR‐49 levels and/or *fmo‐2* expression. Indeed, although loss of *sek‐1* had little effect on hypodermal *fmo‐2p::gfp* expression, it limited the intestinal induction of *fmo‐2p::gfp* by tBOOH and fasting (Figure 4c). Moreover, qPCR analysis revealed that, although the expression of some tBOOH‐induced genes was unaffected, loss of *sek‐1* or downstream *pmk‐1* prevented the full induction of *fmo‐2*,* nlp‐25*, and K05B2.4 mRNA by tBOOH (Figure 4d). However, although *sek‐1* is vital for tBOOH‐induced increases in PMK‐1 phosphorylation (Inoue et al., 2005) (Figure S4e), loss of *sek‐1* did not have a significant impact on tBOOH‐induced increases in the levels or decreased mobility of NHR‐49::GFP (Figure S4f). These data suggest that PMK‐1 activity plays a minor role in the tBOOH‐induced activation of some NHR‐49‐dependent genes, but is not required for tBOOH‐induced increases in NHR‐49.

### 2.6 *nhr‐49* and two NHR‐49‐regulated genes increase stress resistance of wild‐type and long‐lived *glp‐1* mutant worms

**Figure 5:**
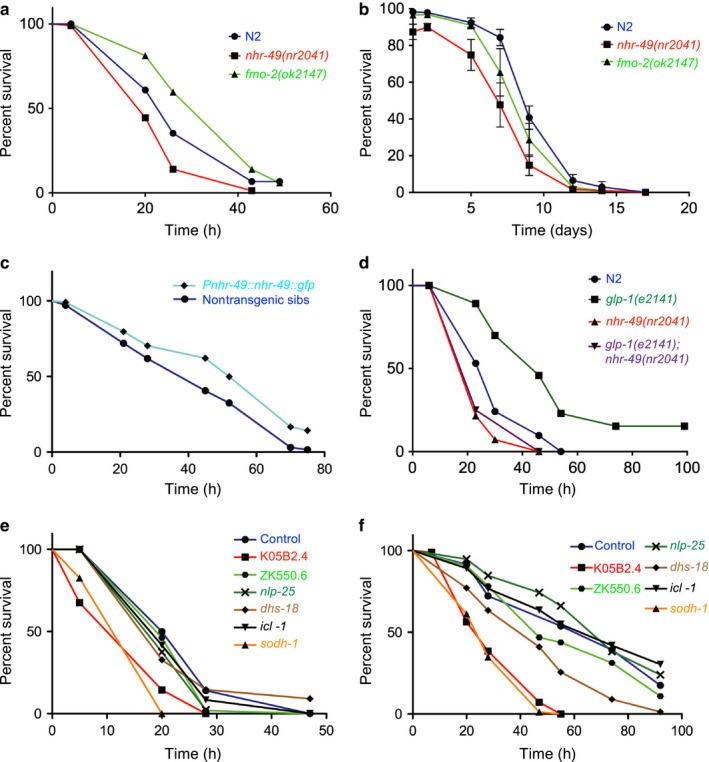
*nhr‐49* is required and sufficient for stress resistance. (a) Survival plots of wild‐type N2, *nhr‐49*(*nr2041*), and *fmo‐2*(*ok2147*) worms on 6 mm tBOOH. Table S6 shows statistics and replicates. (b) Survival of wild‐type N2, *nhr‐49*(*nr2041*), and *fmo‐2*(*ok2147*) worms after L1 fasting over time (*p *<* *.05 vs. N2 for both genotypes, determined by calculating area under the curve). (c) Survival plots of NHR‐49 overexpressing worms (*Pnhr‐49::nhr‐49::gfp*) and nontransgenic siblings, on 6 mm tBOOH. Table S7 shows statistics and replicates. (d) Survival plots of wild‐type N2, *glp‐1*(*e2141*), *nhr‐49*(*nr2041*), and *nhr‐49*(*nr2041*)*; glp‐1*(*e2141*) worms on 6 mm tBOOH. Tables S9 shows statistics and replicates. (e, f) Survival plots of wild‐type (e) and *glp‐1*(*e2141*) (f) worms grown on control RNAi or RNAi clones targeting six different NHR‐49‐regulated genes while exposed to 6 mm tBOOH. Tables S10 and S11 show statistics and replicates

Finally, we tested whether NHR‐49's role in promoting the expression of stress‐responsive genes might be important for survival in stress conditions. We have previously shown that the loss of *nhr‐49* reduces the ability of animals to survive exposure to toxic levels of tBOOH (Goh et al., 2014) (Figure 5a; Table S6). Furthermore, although *fmo‐2* mutant animals were, unexpectedly, more resistant to tBOOH than wild‐type animals (Figure 5a), both *nhr‐49* and *fmo‐2* null mutant L1 larvae were less able to survive and recover from fasting (Figure 5b). Next, we tested whether the elevated basal expression of tBOOH‐responsive genes in the *nhr‐49*(*et13*) and long‐lived *glp‐1*(*e2141*) mutants might be sufficient to increase stress resistance. Indeed, a small, but statistically significant increase was observed in the survival of both NHR‐49 overexpressing (*Pnhr‐49::nhr‐49::GFP*) and *nhr‐49*(*et13*) gof worms exposed to tBOOH (Figures 5c and S5a, Tables S7 and S8). Moreover, long‐lived *glp‐1* mutant worms displayed a substantially increased resistance to tBOOH (Figure 5d) (Steinbaugh et al., 2015), which was abrogated in *nhr‐49*(*nr2041*)*; glp‐1*(*e2141*) double mutants (Figure 5d; Table S9). It is likely that multiple genes contribute to NHR‐49‐dependent increases in stress resistance. Indeed, surprisingly, loss of *fmo‐2* actually increased tBOOH resistance, suggesting that in its absence, other stress‐protective genes may be upregulated (Figure 5a). However, RNAi depletion of two other NHR‐49‐upregulated, tBOOH‐responsive genes (K05B2.4, *sodh‐1*) consistently increased the tBOOH sensitivity of both wild‐type and germline‐less *glp‐1* mutant worms (Figure 5e,f; Tables S10 and S11). In contrast, and in line with our gene expression data (Figure 3), *hlh‐30* mutants were not sensitive to tBOOH (Figure S5b, Table S12). Together, these data suggest that *nhr‐49* plays an important role in promoting the survival of *C. elegans* exposed to organic peroxide or experiencing fasting and identify three NHR‐49‐upregulated genes that are important for resistance to at least one of these conditions.

## 3 DISCUSSION

**Figure 6:**
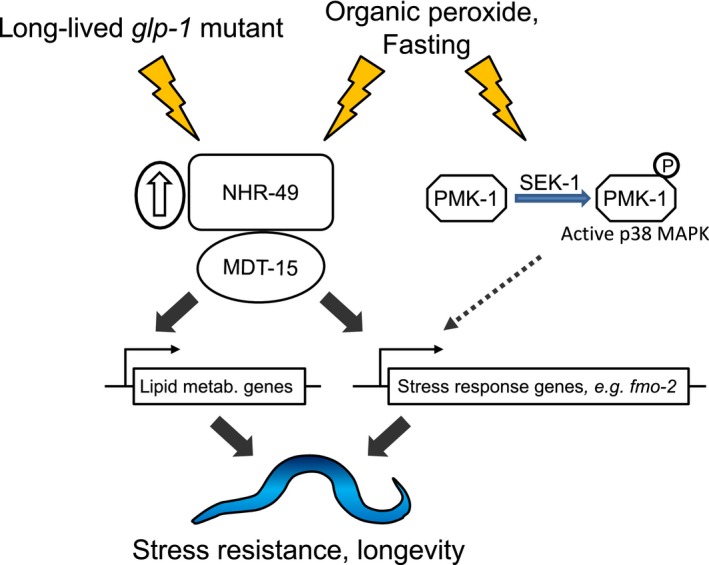
Model for NHR‐49 action in stress defense and longevity

NHR‐49 is a critical regulator of lipid metabolism and longevity (Burkewitz et al., 2015; Chamoli, Singh, Malik & Mukhopadhyay, 2014; Folick et al., 2015; Khan et al., 2013; Ratnappan et al., 2014; Seah et al., 2016), while related nuclear receptors, HNF4 and PPARα, are therapeutic targets that share at least some of these functions in mammals. Here, we show that NHR‐49 is also vital for an adaptive transcriptional response to the organic peroxide tBOOH. This response includes the induction of the flavin‐containing monooxygenase *fmo‐2*, which is important for dietary restriction‐induced longevity and resistance to several stresses (Leiser et al., 2015), and two other genes, K05B2.4 and *sodh‐1*, that we reveal here to be important for resistance to organic peroxide. Moreover, we find that *nhr‐49* is also required for the induction of tBOOH‐induced genes by acute fasting. Collectively, our data suggest that, in addition to its role in regulating lipid metabolism, NHR‐49 participates in a cytoprotective acute stress response program that functions parallel to and independently of SKN‐1/Nrf2 and HLH‐30/TFEB signaling (Blackwell et al., 2015; Lapierre et al., 2013). This raises the possibility that NHR‐49's pro‐longevity function, for example in *glp‐1* mutants (Ratnappan et al., 2014), may involve modulating both lipid metabolism and stress defenses (Figure 6).

NHR‐49 regulates transcription in response to fasting, to drive changes in lipid metabolism (Van Gilst, Hadjivassiliou & Yamamoto, 2005). Here, we report *nhr‐49*‐dependent expression of several fasting‐induced genes with pro‐survival/longevity functions that are not involved in lipid metabolism, including the glyoxylate cycle enzyme *icl‐1*, the oxidoreductase *sodh‐1*, and the flavin‐containing monooxygenase *fmo‐2*. Consistent with these findings, *nhr‐49*‐dependent induction of predicted detoxification genes was observed in another long‐lived state (Chamoli et al., 2014). This was ascribed to NHR‐49‐driven lipid metabolic reprogramming, but our findings suggest that these genes may contribute to NHR‐49's stress‐protective function. Indeed, we show that an NHR‐49‐dependently induced gene, K05B2.4, orthologous to important lipid metabolic enzymes, bile acid‐CoA: amino acid N‐acyltransferase (BAAT) and acyl‐CoA thioesterases (ACOT), is required for resistance to organic peroxide. Thus, by coordinately rewiring lipid and nonlipid metabolic processes, NHR‐49 and MDT‐15 may simultaneously allow robust fasting adaptation and promote stress resistance. If these dual functions are conserved, this has important implications for how metabolic changes, in response to diet, could affect cell/tissue stress defenses in mammals.

Transcriptome profiling showed that NHR‐49 regulates a substantial fraction, but not all of the genes induced by tBOOH in wild‐type; thus, other transcription factors are likely also important for tBOOH‐induced gene expression. Surprisingly, our analysis further revealed a large set of genes that is only weakly induced by tBOOH in wild‐type, but is hyperactivated in *nhr‐49* mutants, suggesting a compensatory response in this strain incapable of mounting a normal tBOOH response (Figure 2b). In the future, it will be interesting to further dissect this response.

MDT‐15 is required as a coactivator for SKN‐1, promoting SKN‐1‐dependent gene expression and/or longevity in several contexts, for example in long‐lived *glp‐1* mutants (Goh et al., 2014; Rogers et al., 2011; Wei & Kenyon, 2016). Thus, *mdt‐15* may promote both NHR‐49 and SKN‐1‐dependent gene expression in germline‐deficient *glp‐1* mutant animals, including different subsets of lipid metabolic and cytoprotective genes. Notably, although both transcription factors require MDT‐15, *nhr‐49* does not activate arsenite‐responsive genes (Figure S2b) and *skn‐1* is dispensable for most tBOOH‐induced gene expression (Oliveira et al., 2009). Thus, NHR‐49 and SKN‐1 are both important in stress conditions and in long‐lived *glp‐1* mutants, but partner with MDT‐15 to coordinate expression of distinct gene sets that may be more important under particular conditions. For example, in long‐lived *daf‐2*/insulin receptor mutants, where *skn‐1* is important, but *nhr‐49* dispensable for the increase in lifespan (Ratnappan et al., 2014; Tullet et al., 2008), the requirement for *mdt‐15* may exclusively reflect MDT‐15's function as a SKN‐1 coregulator (Goh et al., 2014).

Although NHR‐13, NHR‐66, and NHR‐80 heterodimerize with NHR‐49 (Pathare et al., 2012), these three NHRs were not required for tBOOH‐induced gene activation (Figure S2a). In the tBOOH and fasting response, NHR‐49 may instead homodimerize or partner with other NHRs. One candidate is NHR‐62, which is important for lifespan extension in the *eat‐2* model of DR (Heestand et al., 2013). However, although *fmo‐2* is induced by some DR feeding regimens (Leiser et al., 2015), it is not upregulated in *eat‐2* mutants, and *nhr‐49* is dispensable for the increased longevity of this mutant (Heestand et al., 2013). These studies suggest that NHR‐62 and NHR‐49 may act in parallel pro‐longevity pathways that are both regulated by nutrient availability and provide further evidence that different fasting/DR regimes evoke different pro‐longevity transcriptional responses involving different transcriptional regulators to extend lifespan (Greer & Brunet, 2009).

Our discovery that fasting and peroxide stresses activate a similar, NHR‐49‐dependent transcriptional response raised the possibility that in both cases, NHR‐49 might be regulated by a reactive oxygen metabolite. Hydrogen peroxide is likely increased upon fasting, potentially as a by‐product of increased fatty acid oxidation. Although it may cause oxidative damage, peroxide is also an established signaling molecule, regulating the activity of target proteins by oxidizing specific redox‐sensitive protein thiols (Veal, Day & Morgan, 2007). However, using the oxidation status of the peroxide‐sensitive peroxiredoxin‐2 as a readout (Oláhová et al., 2008), we could find no evidence that fasting increases peroxide levels (data not shown). Alternatively, lipid‐derived ligand(s), including oxidized molecules, may increase NHR‐49 activity, with exposure to organic peroxide or fasting both increasing the levels of such a lipid. A recent paper showed that linolenic acid, and an oxidized derivative thereof, regulate lifespan and gene expression in an *nhr‐49*‐dependent fashion (Qi et al., 2017). Although we note that *fmo‐2* was not among the responsive genes (Qi et al., 2017), this nevertheless raises the possibility that similar molecules may be involved in the *nhr‐49*‐dependent stress adaptation mechanisms identified here.

Notably, our data suggest that NHR‐49 activity is increased in response to tBOOH by increasing NHR‐49 protein levels. In contrast to the increased NHR‐49 levels in *glp‐1* mutants, which reflect DAF‐16‐ and TCER‐1‐dependent upregulation of *nhr‐49* mRNA (Ratnappan et al., 2014), our data suggest that this increase does not reflect tBOOH‐induced increases in *nhr‐49* mRNA levels (Figure S4d). It remains to be determined whether this is due to a general increase in NHR‐49 protein stability or specific increases in a particular isoform. However, the tBOOH‐induced increase in levels of a lower mobility NHR‐49 isoform suggests that increases in NHR‐49 levels/activity may involve tBOOH‐induced posttranslational modification(s). We note that NHR‐49 contains several candidate phosphorylation sites, some of which are phosphorylated in vivo (Bodenmiller et al., 2008). Moreover, phosphorylation regulates the activity of functionally related mammalian NHRs, for example PPARα (Burns & Vanden Heuvel, 2007). Hence, it is possible that tBOOH‐induced phosphorylation might stabilize NHR‐49 or direct its activity toward activating the expression of *fmo‐2* and other stress‐induced genes. The p38 MAPK, PMK‐1, plays an important role in defending against oxidative stress and infectious microbes. For example, PMK‐1‐dependent phosphorylation is vital for the stress‐induced nuclear accumulation of SKN‐1 and activation of SKN‐1‐dependent gene expression (Inoue et al., 2005). Moreover, PMK‐1 is rapidly activated by phosphorylation by the SEK‐1 MAPKK in response to tBOOH (Inoue et al., 2005). However, although loss of *sek‐1* and *pmk‐1* reduced the tBOOH‐induced expression of *fmo‐2* and two other *nhr‐49‐*dependent genes, tBOOH‐induced increases in NHR‐49 levels occurred in the absence of *sek‐1*. This suggests that, although SEK‐1 and PMK‐1 may act in parallel with NHR‐49 to promote tBOOH‐induced gene expression, other mechanisms, potentially involving other kinases, are responsible for increases in NHR‐49 levels.

In summary, we show that NHR‐49 acts with MDT‐15 to implement a stress‐protective transcriptional program in response to organic peroxide, fasting, and in long‐lived *glp‐1* mutant worms. Future experiments will determine how fasting and oxidative stress regulate NHR‐49 and/or MDT‐15 to coordinate both changes in lipid metabolism and other protective adaptive changes in host metabolism. If related NHRs, PPARα and HNF4, play similar roles in protecting mammalian cells under stress conditions, then this may have implications for efforts to therapeutically target these NHRs to treat age‐related diseases such as metabolic diseases and cancer.

## 4 EXPERIMENTAL PROCEDURES

### 4.1 Nematode strains and growth conditions

We cultured *C. elegans* strains using standard techniques. To avoid background effects, each mutant was crossed into our N2 strain; original mutants were backcrossed to N2 at least six times. *E. coli* OP50 was the food source in all qPCR and RNA‐seq experiments except in Figure 3b, all population stress resistance experiments (tBOOH and starvation assays; Figure 5), and for the initial characterization of the *fmo‐2p::gfp* reporter (Figures 1b and S1b); for all other experiments, we used *E. coli* HT115 as food source. All experiments were carried out at 20°C, except experiments with strains harboring the *glp‐1*(*e2141*) mutation; these strains and pertinent controls were grown at 25°C until the L4 stage to induce germline loss, and then downshifted to 20°C. Worm strains used in this study are listed in Table S13.

We used standard genetic crossing techniques to attempt to construct the *nhr‐49*(*nr2041*)*; hlh‐30*(*tm1978*) double mutant; genotyping the clonal progeny of 100 individual candidate F2 worms did not reveal any homozygous double mutants, suggesting that these null mutations cause synthetic lethality.

For experiments on media containing exogenous stressors, sodium meta‐arsenite (Sigma 71287) and tert‐butyl hydroperoxide solution (tBOOH; Sigma 458139), DTT, and H_2_O_2_ were added at indicated concentrations. Plates containing tBOOH were made fresh on the day of use. For the RNAi candidate screen, we used 10 mm tBOOH and a 3‐hr exposure as these conditions resulted in strong and uniform GFP induction throughout the worm population. For qPCR and RNA‐seq experiments, we used 7.5 mm tBOOH exposure for 4 hr, consistent with our previous study (Goh et al., 2014). For stress resistance assays, we found that resolution of population death events was improved using 6 mm tBOOH and hence used this concentration.

For synchronized worm growths, bleached embryos were hatched overnight on unseeded NGM plates, or 20–25 L4 stage worms were transferred and maintained on feeding plates until the population reached a synchronized halted development at L1 stage via short‐term fasting (12–24 hr). Synchronized L1 stage larvae were subsequently transferred to feeding plates, seeded with OP50 or HT115 containing the indicated RNAi vector, and then grown to the desired stage.

### 4.2 Feeding RNA interference

RNAi was performed on nematode growth media (NGM) plates supplemented with either 25 μg/ml carbenicillin or 100 μg/ml ampicillin, 1 mm IPTG, and 12.5 μg/ml tetracycline and seeded with appropriate HT115 RNAi bacteria. The RNAi clones were from the Ahringer (Source BioScience) or Vidal library except those listed in Table S14.

### 4.3 Construction of the *fmo‐2p::gfp* transgenic reporter strain

A 1584‐bp genomic fragment of the putative *fmo‐2* promoter was PCR‐amplified with the primers (P*fmo‐2*Fwd = 5′CCCATGACTGCAGGTGCAGAAGTAAGATATTGCA and P*fmo‐2*Rev = 5′CCCGCTACCCGGGACGGTAATCCAGAAGCACCA, pPD95.67 + P*fmo‐2* Rev = 5′CGGTACCTCCCTCCAAGGGTCCTC; Pst1 and Cfr91 sites used for cloning underlined), sequenced, and cloned into pPD95.67 using Pst1 and Cfr91 sites. This generates a transgene containing 1,536 bp of DNA upstream of the *fmo‐2* ORF and the first 16 codons of FMO‐2 fused in frame to GFP containing a nuclear localization signal (pPD95.67 + *fmo‐2p*). We then microinjected into N2 worms 50 ng/μl pPD95.67 + *fmo‐2p*, 100 ng/μl of pRF4 *rol‐6*(*su1006*) co‐injection marker, and 50 ng/μl pBluescript carrier DNA. Two independently generated lines were selected using the roller phenotype. Both lines showed similar expression patterns and induction by tBOOH (data not shown).

### 4.4 RNA isolation and qPCR analysis

RNA isolation was performed essentially as described (Goh et al., 2014). 2 μg total RNA were used to generate cDNA with Superscript II reverse transcriptase (Invitrogen 18064‐014), random primers (Invitrogen 48190‐011), dNTPs (Fermentas R0186), and RNAseOUT (Invitrogen 10777‐019). qPCR was performed in 30 μl reactions using Fast SYBR Master Mix (Life Technologies 4385612) and an Applied Biosystems StepOnePlus machine. We analyzed data with the ΔΔCt method. For each sample, we calculated normalization factors by averaging the (sample expression)/(reference expression) ratios of three normalization genes, *act‐1*,* tba‐1*, and *ubc‐2* (except without *tba‐1* in *glp‐1* mutants, which display inherently low *tba‐1* levels). The reference sample was *control*(*RNAi*), WT, or untreated, as appropriate, and all data are represented as mean ± SEM. We used unpaired Student's *t* tests to calculate statistical significance of gene expression changes and corrected for multiple comparisons using the Holm–Sidak method. Primers were tested on serial cDNA dilutions and analyzed for PCR efficiency prior to use. All data originate from three or more independent biological repeats, and each experiment was conducted in technical triplicate. Sequences of primers used are listed in Table S15.

### 4.5 Analysis of gene expression overlaps

Gene expression overlaps were identified using the Microsoft Excel 2011 ISNA (MATCH(lookup_value, lookup_array, [match_type])) function. Statistical significance was calculated using Fisher's exact test from the R Stats Package in RStudio version 0.98. We compared sets of genes regulated in fasted worms (Uno et al., 2013), tBOOH‐treated worms (Oliveira et al., 2009), and *glp‐1*(*bn18*) mutants (Steinbaugh et al., 2015).

### 4.6 RNA‐seq

L4 stage N2 and *nhr‐49*(*nr2041*) worms were exposed to 7.5 mm tBOOH for 4 hr. RNA was extracted with TRIzol and ribosomal RNA removed using the RiboGone–Mammalian—Low Input Ribosomal RNA Removal Kit for Human, Mouse and Rat Samples (Takara #634847). Ribosomal RNA depletion and RNA integrity were verified on a Bioanalyzer with the RNA 6000 Pico Kit (Agilent #5067‐1513). We constructed cDNA libraries with the SMARTer Universal Low Input RNA Kit (Takara #634900) and measured adapter‐ligated concentrations with the High Sensitivity DNA Analysis Kit (Agilent #5067‐4626) and by qPCR standard‐curve quantitation using the KAPA Library Quantification Kit Illumina Platforms (KK4824). Next‐generation sequencing was performed on an Illumina NextSeq 500 using 76 bp x2 paired‐end reads, using the NextSeq 500/550 High Output Kit v2 (150 cycles). Single‐end reads were aligned to the *C. elegans* UCSC ce10 reference genome using an RNA‐seq alignment app from Illumina, which uses STAR aligner to map reads. Then, we used edgeR (Robinson, McCarthy & Smyth, 2010) to convert raw gene counts into counts per million (CPM) and log2‐counts per million (log‐CPM). Weakly expressed genes were removed by only including genes with counts per million (CPM) >1 across two libraries. Gene expression distributions were normalized by the method of trimmed mean of M‐values (TMM) (Robinson & Oshlack, 2010). We used the edgeR exact test to test for differential expression between different conditions. We searched for differentially expressed genes using Top Tags in edgeR with log‐fold change of (+2) and FDR <0.05 cutoffs and identified 250 genes that were induced by tBOOH in wild‐type worms. We then obtained log‐CPM values for genes in this set from all eight libraries, corresponding to two replicates of wild‐type worms and *nhr‐49*(*nr2041*) mutants −/+ tBOOH (Tables S3 and S4), and used FDR <0.05 cutoffs to define NHR‐49‐dependent genes within this set (Tables S3 and S4). We used the heatmap.2 function to generate a heatmap of these 250 genes using log‐CPM values. Expression across each gene was scaled to generate a *z*‐score. In Figure 2b, genes were ordered from highest to lowest difference in *z*‐score between N2 + tBOOH and *nhr‐49 *+* *tBOOH samples. RNA‐seq raw data have been deposited at Gene Expression Omnibus (GSE107799).

### 4.7 DIC and fluorescence microscopy

Worms were transferred onto M9 buffer containing 0.06% levamisole (Sigma L9756) or sodium azide for immobilization on 2%–2.5% (w/v) agarose pads for microscopy. We captured images either on a CoolSnap HQ camera (Photometrics) attached to a Zeiss Axioplan 2 compound microscope, followed by MetaMorph Imaging Software with Autoquant 3D digital deconvolution; or using a Zeiss Axioskop 2 compound microscope, an Axiocam, and AxioVision software (version 3.1.2.1).

### 4.8 Stress resistance assays

To quantify tBOOH resistance in a population, animals were grown to the late L4 stage and then transferred to plates containing 6 mm tBOOH seeded with heat‐inactivated OP50. Animals that crawled off the plate or died due to rupturing or internal hatching were censored. We used GraphPad Prism 6 to generate survival curves and calculated statistical significance using the log‐rank (Mantel–Cox) test. For each individual experiment, we performed three to four independent biological replicates, and one repeat is shown in a figure panel; for this and all other repeats, experimental parameters and statistics are listed in the Supplementary information, as indicated in figure legends. For experiments with *nhr‐49p::nhr‐49::gfp* and *nhr‐49*(*et13*) worms, we additionally performed Cox's regression analysis to test the statistical significance of differences between the survival of transgenic/mutant and control worms across all biological repeats (Tables S7 and S8).

Starvation survival assays were adapted from (Lee & Ashrafi, 2008). Worms were maintained for at least two generations in a nonstarved state and synchronized to obtain day 1 adults. Adults were then bleached to obtain embryos that were hatched in S‐basal medium without cholesterol overnight on a rotator. Synchronized L1s were transferred to S‐basal medium without cholesterol containing an antibiotic–antimycotic mix (Gibco 15240062) at a concentration of ~1 worm/μl. To assess viability, 200–300 animals were transferred to seeded NGM plates every 2–3 days and assessed for growth to the L4 stage after 48 hr. Average survival and standard error of four independent biological repeats are shown in Figure 5b; statistical significance was calculated using area under the curve and Student's *t* test.

### 4.9 SDS–PAGE and immunoblotting

#### 4.9.1 Analysis of NHR‐49 + GFP levels

A total of 2,000–3,000 synchronized young adult worms, maintained on *E. coli* HT115‐seeded plates, were washed off (Figures 4b and S4b,c), or 50 synchronized RFP+ or control young adult worms were picked off (Figure S4e,f) *E. coli* HT115‐seeded plates into M9 or M9 containing the indicated stress agent (Figures 4 and S4e,f). After the indicated time, an equal volume of ice‐cold 20% (v/v) tri‐chloro acetic acid (TCA; Sigma T6399) was added to precipitate proteins. Pelleted proteins were washed with ice‐cold acetone before being air‐dried and resuspended in TCA buffer (1.0% SDS (v/v), 100 mm Tris–HCl pH 8.0, 1.0 mm EDTA (Sigma E6758) + 20 mm N‐ethylmaleimide (Sigma E3876)). The suspension was then incubated for 30 min at 25°C followed by 5 min at 30°C. Insoluble materials were pelleted by centrifugation. The concentration of denatured proteins in the supernatant was determined using the BCA Protein Assay Kit (Thermo Scientific 23225), and equal amounts of protein were separated by SDS–PAGE and immunoblotted using the appropriate antibodies in conjunction with the ECL Plus Western Blotting Substrate (Thermo Scientific 32132) and a Typhoon Phosphoimager. NHR‐49::GFP levels were determined relative to tubulin to account for any differences in loading.

#### 4.9.2 Analysis of PMK‐1 phosphorylation

A total of 3,000 synchronized late L4 animals were washed from plates with M9 or M9 containing 10 mm tBOOH, incubated for 10 min with gentle agitation, and then harvested by centrifugation and levels of PMK‐1 phosphorylation determined as described previously (Oláhová et al., 2008).

### 4.10 Antibodies

All antibodies were diluted in 1× Tris buffer saline Tween 20 (TBST; 20.0 mm Tris–HCl pH 7.6, 137 mm NaCl, 0.1% Tween 20 (v/v) containing 5% bovine serum album). Anti‐GFP (A6455, Life Technologies), anti‐phosphop38 antibodies (New England Biolabs), and anti‐β‐tubulin (E7, Developmental Studies Hybridoma Bank University of Iowa) antibodies were used at 1:1,000 dilutions. Horseradish peroxidase (HRP)‐conjugated secondary anti‐mouse IgG and anti‐rabbit IgG antibodies were used at 1:2,000.

## CONFLICT OF INTEREST

None declared.

## AUTHORS' CONTRIBUTION

EAV and ST conceptualized the study; GYSG, JJW, EAV, KRSD, FB, RL, and KL investigated the study; ST and EAV wrote the original draft; ST, EAV, GG, and JJW wrote the manuscript and helped in reviewing and editing the manuscript; EAV and ST acquired funding; EAV and ST supervised the experiments.

## References

[acel12743-bib-0001] Bennett, C. F. , Kwon, J. J. , Chen, C. , Russell, J. , Acosta, K. , Burnaevskiy, N. , … Kaeberlein, M. (2017). Transaldolase inhibition impairs mitochondrial respiration and induces a starvation‐like longevity response in *Caenorhabditis elegans*. C. T. Murphy, ed. PLoS Genetics, 13, e1006695 10.1371/journal.pgen.1006695 28355222 PMC5389855

[acel12743-bib-0002] Blackwell, T. K. , Steinbaugh, M. J. , Hourihan, J. M. , Ewald, C. Y. , & Isik, M. (2015). SKN‐1/Nrf, stress responses, and aging in *Caenorhabditis elegans* . Free Radical Biology and Medicine, 88, 290–301. 10.1016/j.freeradbiomed.2015.06.008 26232625 PMC4809198

[acel12743-bib-0003] Bodenmiller, B. , Campbell, D. , Gerrits, B. , Lam, H. , Jovanovic, M. , Picotti, P. , … Aebersold, R. (2008). PhosphoPep–a database of protein phosphorylation sites in model organisms. Nature Biotechnology, 26, 1339–1340. 10.1038/nbt1208-1339 PMC2743685 19060867

[acel12743-bib-0004] Burkewitz, K. , Morantte, I. , Weir, H. J. M. , Yeo, R. , Zhang, Y. , Huynh, F. K. , … Mair, W. B. (2015). Neuronal CRTC‐1 governs systemic mitochondrial metabolism and lifespan via a catecholamine signal. Cell, 160, 842–855. 10.1016/j.cell.2015.02.004 25723162 PMC4392909

[acel12743-bib-0005] Burns, K. A. , & Vanden Heuvel, J. P. (2007). Modulation of PPAR activity via phosphorylation. Biochimica et Biophysica Acta, 1771, 952–960. 10.1016/j.bbalip.2007.04.018 17560826 PMC2712836

[acel12743-bib-0006] Chamoli, M. , Singh, A. , Malik, Y. , & Mukhopadhyay, A. (2014). A novel kinase regulates dietary restriction‐mediated longevity in *Caenorhabditis elegans* . Aging Cell, 13, 641–655. 10.1111/acel.12218 24655420 PMC4326946

[acel12743-bib-0007] Folick, A. , Oakley, H. D. , Yu, Y. , Armstrong, E. H. , Kumari, M. , Sanor, L. , … Wang, M. C. (2015). Aging. Lysosomal signaling molecules regulate longevity in *Caenorhabditis elegans* . Science, 347, 83–86. 10.1126/science.1258857 25554789 PMC4425353

[acel12743-bib-0008] Goh, G. Y. S. , Martelli, K. L. , Parhar, K. S. , Kwong, A. W. L. , Wong, M. A. , Mah, A. , … Taubert, S. (2014). The conserved mediator subunit MDT‐15 is required for oxidative stress responses in *Caenorhabditis elegans* . Aging Cell, 13, 70–79. 10.1111/acel.12154 23957350 PMC4326869

[acel12743-bib-0009] Greer, E. L. , & Brunet, A. (2009). Different dietary restriction regimens extend lifespan by both independent and overlapping genetic pathways in *Caenorhabditis elegans* . Aging Cell, 8, 113–127. 10.1111/j.1474-9726.2009.00459.x 19239417 PMC2680339

[acel12743-bib-0010] Heestand, B. N. , Shen, Y. , Liu, W. , Magner, D. B. , Storm, N. , Meharg, C. , … Antebi, A. (2013). Dietary restriction induced longevity is mediated by nuclear receptor NHR‐62 in *Caenorhabditis elegans* . PLoS Genetics, 9, e1003651 10.1371/journal.pgen.1003651 23935515 PMC3723528

[acel12743-bib-0011] Hekimi, S. , Lapointe, J. , & Wen, Y. (2011). Taking a “good look” at free radicals in the aging process. Trends in Cell Biology, 21, 569–576. 10.1016/j.tcb.2011.06.008 21824781 PMC4074523

[acel12743-bib-0012] Hetz, C. , Chevet, E. , & Harding, H. P. (2013). Targeting the unfolded protein response in disease. Nature Reviews. Drug Discovery, 12, 703–719. 10.1038/nrd3976 23989796

[acel12743-bib-0013] Inoue, H. , Hisamoto, N. , An, J. H. , Oliveira, R. P. , Nishida, E. , Blackwell, T. K. , & Matsumoto, K. (2005). The *Caenorhabditis elegans* p38 MAPK pathway regulates nuclear localization of the transcription factor SKN‐1 in oxidative stress response. Genes & Development, 19, 2278–2283. 10.1101/gad.1324805 16166371 PMC1240035

[acel12743-bib-0014] Khan, M. H. , Ligon, M. , Hussey, L. R. , Hufnal, B. , Farber, R. , Munkácsy, E. , … Rea, S. L. (2013). TAF‐4 is required for the life extension of isp‐1, clk‐1 and tpk‐1 Mit mutants. Aging, 5, 741–758. https://doi.org/10.18632/aging.100604 24107417 10.18632/aging.100604 PMC3838777

[acel12743-bib-0015] Lapierre, L. R. , De Magalhaes Filho, C. D. , McQuary, P. R. , Chu, C.‐C. , Visvikis, O. , Chang, J. T. , … Hansen, M. (2013). The TFEB orthologue HLH‐30 regulates autophagy and modulates longevity in *Caenorhabditis elegans* . Nature Communications, 4, 2267. 10.1038/ncomms3267 PMC3866206 23925298

[acel12743-bib-0016] Lee, B. H. , & Ashrafi, K. (2008). A TRPV channel modulates *Caenorhabditis elegans* neurosecretion, larval starvation survival, and adult lifespan. PLoS Genetics, 4, e1000213. 18846209 10.1371/journal.pgen.1000213 PMC2556084

[acel12743-bib-0017] Lee, K. , Goh, G. Y. S. , Wong, M. A. , Klassen, T. L. , & Taubert, S. (2016). Gain‐of‐function alleles in *Caenorhabditis elegans* nuclear hormone receptor nhr‐49 are functionally distinct. PLoS ONE, 11, e0162708 10.1371/journal.pone.0162708 27618178 PMC5019492

[acel12743-bib-0018] Leiser, S. F. , Miller, H. , Rossner, R. , Fletcher, M. , Leonard, A. , Primitivo, M. , … Kaeberlein, M. (2015). Cell nonautonomous activation of flavin‐containing monooxygenase promotes longevity and health span. Science, 350, 1375–1378. 10.1126/science.aac9257 26586189 PMC4801033

[acel12743-bib-0019] Leprivier, G. , Rotblat, B. , Khan, D. , Jan, E. , & Sorensen, P. H. (2015). Stress‐mediated translational control in cancer cells. Biochimica et Biophysica Acta, 1849, 845–860. 10.1016/j.bbagrm.2014.11.002 25464034

[acel12743-bib-0020] Lin, M. T. , & Beal, M. F. (2006). Mitochondrial dysfunction and oxidative stress in neurodegenerative diseases. Nature, 443, 787–795. 10.1038/nature05292 17051205

[acel12743-bib-0021] Miranda‐Vizuete, A. , & Veal, E. A. (2016). *Caenorhabditis elegans* as a model for understanding ROS function in physiology and disease. Redox Biology, 11, 708–714. 28193593 10.1016/j.redox.2016.12.020 PMC5304259

[acel12743-bib-0022] Nakamura, S. , Karalay, Ö. , Jäger, P. S. , Horikawa, M. , Klein, C. , Nakamura, K. , … Antebi, A. (2016). Mondo complexes regulate TFEB via TOR inhibition to promote longevity in response to gonadal signals. Nature Communications, 7, 10944 10.1038/ncomms10944 PMC4804169 27001890

[acel12743-bib-0023] Oláhová, M. , Taylor, S. R. , Khazaipoul, S. , Wang, J. , Morgan, B. A. , Matsumoto, K. , … Veal, E. A. (2008). A redox‐sensitive peroxiredoxin that is important for longevity has tissue‐ and stress‐specific roles in stress resistance. Proceedings of the National Academy of Sciences of the United States of America, 105, 19839–19844. 10.1073/pnas.0805507105 19064914 PMC2604961

[acel12743-bib-0024] Oliveira, R. P. , Abate, J. P. , Dilks, K. , Landis, J. , Ashraf, J. , Murphy, C. T. , & Blackwell, T. K. (2009). Condition‐adapted stress and longevity gene regulation by *Caenorhabditis elegans* SKN‐1/Nrf. Aging Cell, 8, 524–541. 10.1111/j.1474-9726.2009.00501.x 19575768 PMC2776707

[acel12743-bib-0025] O'Rourke, E. J. , & Ruvkun, G. (2013). MXL‐3 and HLH‐30 transcriptionally link lipolysis and autophagy to nutrient availability. Nature Cell Biology, 15, 668–676. 10.1038/ncb2741 23604316 PMC3723461

[acel12743-bib-0026] Pathare, P. P. , Lin, A. , Bornfeldt, K. E. , Taubert, S. , & Van Gilst, M. R. (2012). Coordinate regulation of lipid metabolism by novel nuclear receptor partnerships K. Ashrafi, ed. PLoS Genetics, 8, e1002645 10.1371/journal.pgen.1002645 22511885 PMC3325191

[acel12743-bib-0027] Powell‐Coffman, J. A. (2010). Hypoxia signaling and resistance in *Caenorhabditis elegans* . Trends in Endocrinology and Metabolism, 21, 435–440. 10.1016/j.tem.2010.02.006 20335046

[acel12743-bib-0028] Qi, W. , Gutierrez, G. E. , Gao, X. , Dixon, H. , McDonough, J. A. , Marini, A. M. , & Fisher, A. L. (2017). The ω‐3 fatty acid α‐linolenic acid extends *Caenorhabditis elegans* lifespan via NHR‐49/PPARα and oxidation to oxylipins. Aging Cell, 16, 1125–1135. 10.1111/acel.12651 28772063 PMC5595674

[acel12743-bib-0029] Rankin, E. B. , & Giaccia, A. J. (2016). Hypoxic control of metastasis. Science, 352, 175–180. 10.1126/science.aaf4405 27124451 PMC4898055

[acel12743-bib-0030] Ratnappan, R. , Amrit, F. R. G. , Chen, S.‐W. , Gill, H. , Holden, K. , Ward, J. , … Ghazi, A. (2014). Germline signals deploy NHR‐49 to modulate fatty‐acid β‐oxidation and desaturation in somatic tissues of *Caenorhabditis elegans* . PLoS Genetics, 10, e1004829 10.1371/journal.pgen.1004829 25474470 PMC4256272

[acel12743-bib-0031] Robinson, M. D. , McCarthy, D. J. , & Smyth, G. K. (2010). edgeR: A Bioconductor package for differential expression analysis of digital gene expression data. Bioinformatics, 26, 139–140. 10.1093/bioinformatics/btp616 19910308 PMC2796818

[acel12743-bib-0032] Robinson, M. D. , & Oshlack, A. (2010). A scaling normalization method for differential expression analysis of RNA‐seq data. Genome Biology, 11, R25 10.1186/gb-2010-11-3-r25 20196867 PMC2864565

[acel12743-bib-0033] Rogers, A. N. , Chen, D. , Mccoll, G. , Czerwieniec, G. , Felkey, K. , Gibson, B. W. , … Kapahi, P. (2011). Life span extension via eIF4G inhibition is mediated by posttranscriptional remodeling of stress response gene expression in *Caenorhabditis elegans* . Cell Metabolism, 14, 55–66. 10.1016/j.cmet.2011.05.010 21723504 PMC3220185

[acel12743-bib-0034] Seah, N. E. , De Magalhaes Filho, C. D. , Petrashen, A. P. , Henderson, H. R. , Laguer, J. , Gonzalez, J. , … Lapierre, L. R. (2016). Autophagy‐mediated longevity is modulated by lipoprotein biogenesis. Autophagy, 12, 261–272. 10.1080/15548627.2015.1127464 26671266 PMC4836030

[acel12743-bib-0035] Shore, D. E. , & Ruvkun, G. (2013). A cytoprotective perspective on longevity regulation. Trends in Cell Biology, 23, 409–420. 10.1016/j.tcb.2013.04.007 23726168 PMC4057428

[acel12743-bib-0036] Steinbaugh, M. J. , Narasimhan, S. D. , Robida‐Stubbs, S. , Moronetti Mazzeo, L. E. , Dreyfuss, J. M. , Hourihan, J. M. , … Blackwell, T. K. (2015). Lipid‐mediated regulation of SKN‐1/Nrf in response to germ cell absence. Elife, 4, e07836. 10.7554/eLife.07836 PMC4541496 26196144

[acel12743-bib-0037] Taubert, S. , Hansen, M. , Van Gilst, M. R. , Cooper, S. B. , & Yamamoto, K. R. (2008). The mediator subunit MDT‐15 confers metabolic adaptation to ingested material. PLoS Genetics, 4, e1000021. 18454197 10.1371/journal.pgen.1000021 PMC2265483

[acel12743-bib-0038] Taubert, S. , Van Gilst, M. R. , Hansen, M. , & Yamamoto, K. R. (2006). A mediator subunit, MDT‐15, integrates regulation of fatty acid metabolism by NHR‐49‐dependent and ‐independent pathways in *Caenorhabditis elegans* . Genes & Development, 20, 1137–1149. 10.1101/gad.1395406 16651656 PMC1472473

[acel12743-bib-0039] Tullet, J. M. A. , Hertweck, M. , An, J. H. , Baker, J. , Hwang, J. Y. , Liu, S. , … Blackwell, T. K. (2008). Direct inhibition of the longevity‐promoting factor SKN‐1 by insulin‐like signaling in *Caenorhabditis elegans* . Cell, 132, 1025–1038. 10.1016/j.cell.2008.01.030 18358814 PMC2367249

[acel12743-bib-0040] Uno, M. , Honjoh, S. , Matsuda, M. , Hoshikawa, H. , Kishimoto, S. , Yamamoto, T. , … Nishida, E. (2013). A fasting‐responsive signaling pathway that extends life span in *Caenorhabditis elegans* . Cell Reports, 3, 79–91. 10.1016/j.celrep.2012.12.018 23352664

[acel12743-bib-0041] Van Gilst, M. R. , Hadjivassiliou, H. , Jolly, A. , & Yamamoto, K. R. (2005). Nuclear hormone receptor NHR‐49 controls fat consumption and fatty acid composition in *Caenorhabditis elegans* . PLoS Biology, 3, e53 10.1371/journal.pbio.0030053 15719061 PMC547972

[acel12743-bib-0042] Van Gilst, M. R. , Hadjivassiliou, H. , & Yamamoto, K. R. (2005). A *Caenorhabditis elegans* nutrient response system partially dependent on nuclear receptor NHR‐49. Proceedings of the National Academy of Sciences of the United States of America, 102, 13496–13501. 10.1073/pnas.0506234102 16157872 PMC1201344

[acel12743-bib-0043] Veal, E. A. , Day, A. M. , & Morgan, B. A. (2007). Hydrogen peroxide sensing and signaling. Molecular Cell, 26, 1–14. 10.1016/j.molcel.2007.03.016 17434122

[acel12743-bib-0044] Wei, Y. , & Kenyon, C. (2016). Roles for ROS and hydrogen sulfide in the longevity response to germline loss in *Caenorhabditis elegans* . Proceedings of the National Academy of Sciences of the United States of America, 113, E2832–E2841. 10.1073/pnas.1524727113 27140632 PMC4878494

[acel12743-bib-0045] Wu, C.‐W. , Deonarine, A. , Przybysz, A. , Strange, K. , & Choe, K. P. (2016). The Skp1 homologs SKR‐1/2 are required for the *Caenorhabditis elegans* SKN‐1 antioxidant/detoxification response independently of p38 MAPK. PLoS Genetics, 12, e1006361 10.1371/journal.pgen.1006361 27776126 PMC5077136

