# Supplementary material for: NHR‐49/HNF4 integrates regulation of fatty acid metabolism with a protective transcriptional response to oxidative stress and fasting
Source: Aging Cell. 2018 Mar 5;17(3):e12743. doi: 10.1111/acel.12743 (PMC5946062; doi:10.1111/acel.12743)
Supplement: Supplementary file 1 [file ACEL-17-e12743-s001.pdf]

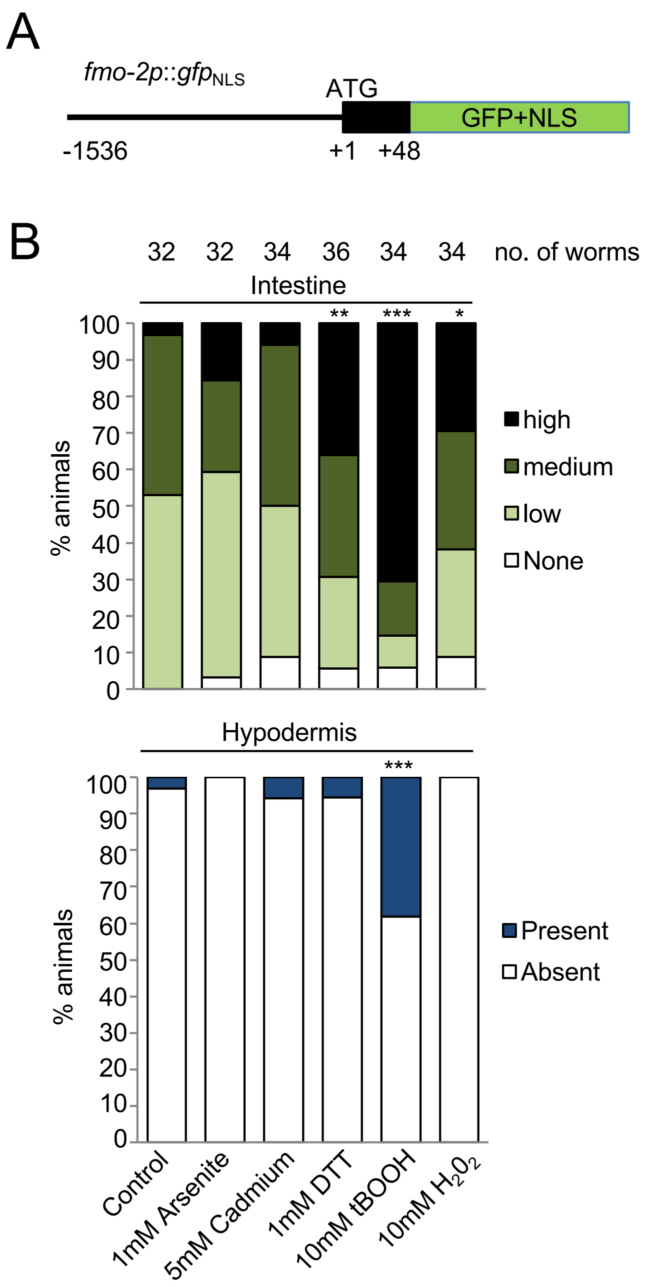

Fig. S1. Transcriptional regulation of *fmo-2* in response to stress.
[A] Scheme of the *fmo-2p::gfp* reporter. The promoter includes 1.5kb upstream of the *fmo-2* open reading frame and the first 48bp downstream of the ATG. NLS=nuclear localization signal.
[B] Bar graphs show the quantification of intestinal and hypodermal fluorescence of *fmo-2p::gfp* in control animals or animals exposed for 180min to arsenite, cadmium, DTT, tBOOH or H_2O_2 at the indicated concentrations. Quantification is based on the number of visible GFP-positive nuclei in each worm. Between 32 and 36 animals were scored for each group, as indicated. Statistically significant differences between control and treated animals are shown: *=p<0.05, **=p<0.005, and ***=p<0.0005 (determined by Chi-squared test).

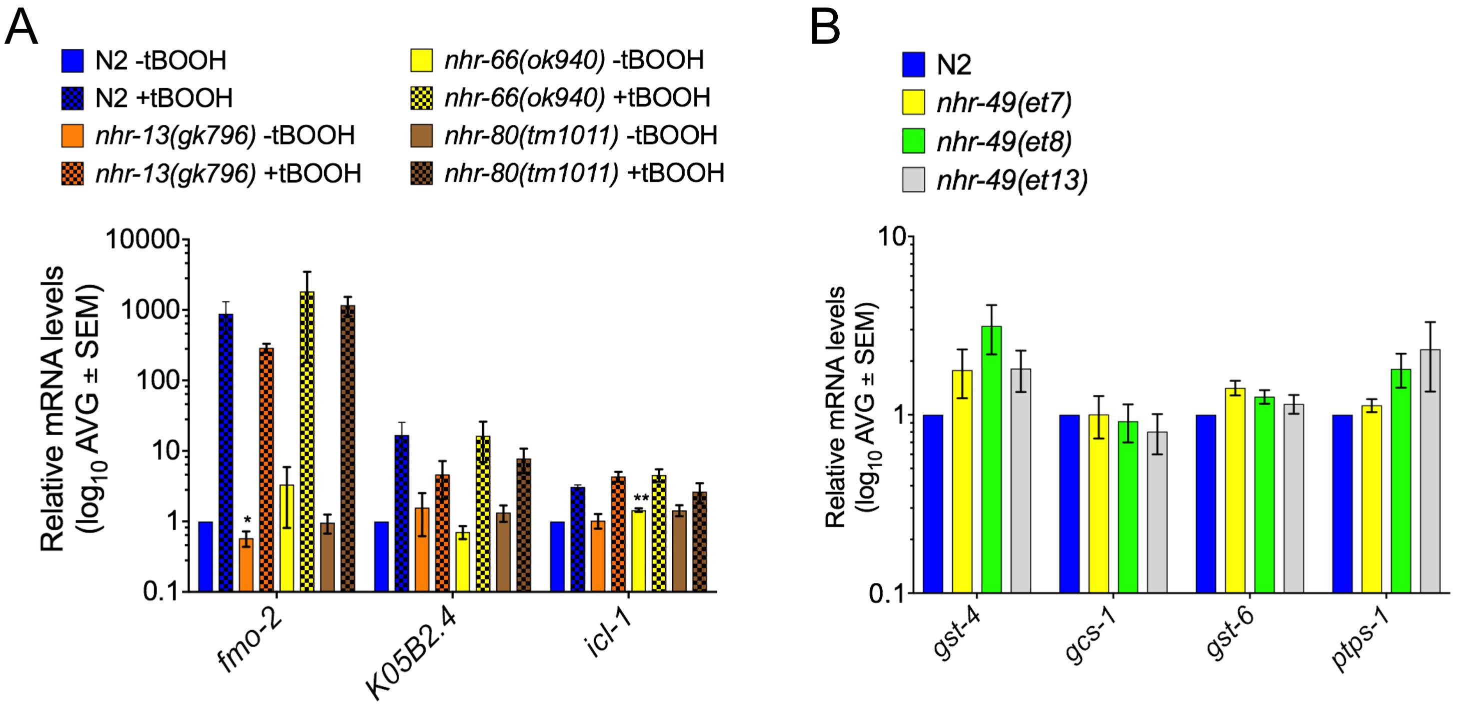

Fig. S2. Regulation of stress response genes by NHR-49 and its NHR partners.

[A] Fold changes of mRNA levels (relative to N2 untreated worms) in L4 stage N2, *nhr-13(gk796)*, *nhr-66(ok940)*, and *nhr-80(tm1011)* worms treated with 7.5 mM tBOOH for 4 hours (n≥3). Error bars represent SEM. Gene expression levels differ significantly from N2 untreated worms: *=p<0.05 and **=p<0.01 (as determined by Student's t-test).

[B] mRNA fold changes (relative to untreated N2 worms) of arsenite-responsive genes in N2 and *nhr-49* GOF mutants (n=3). No significant differences in expression were identified (as determined by Student's t-test).

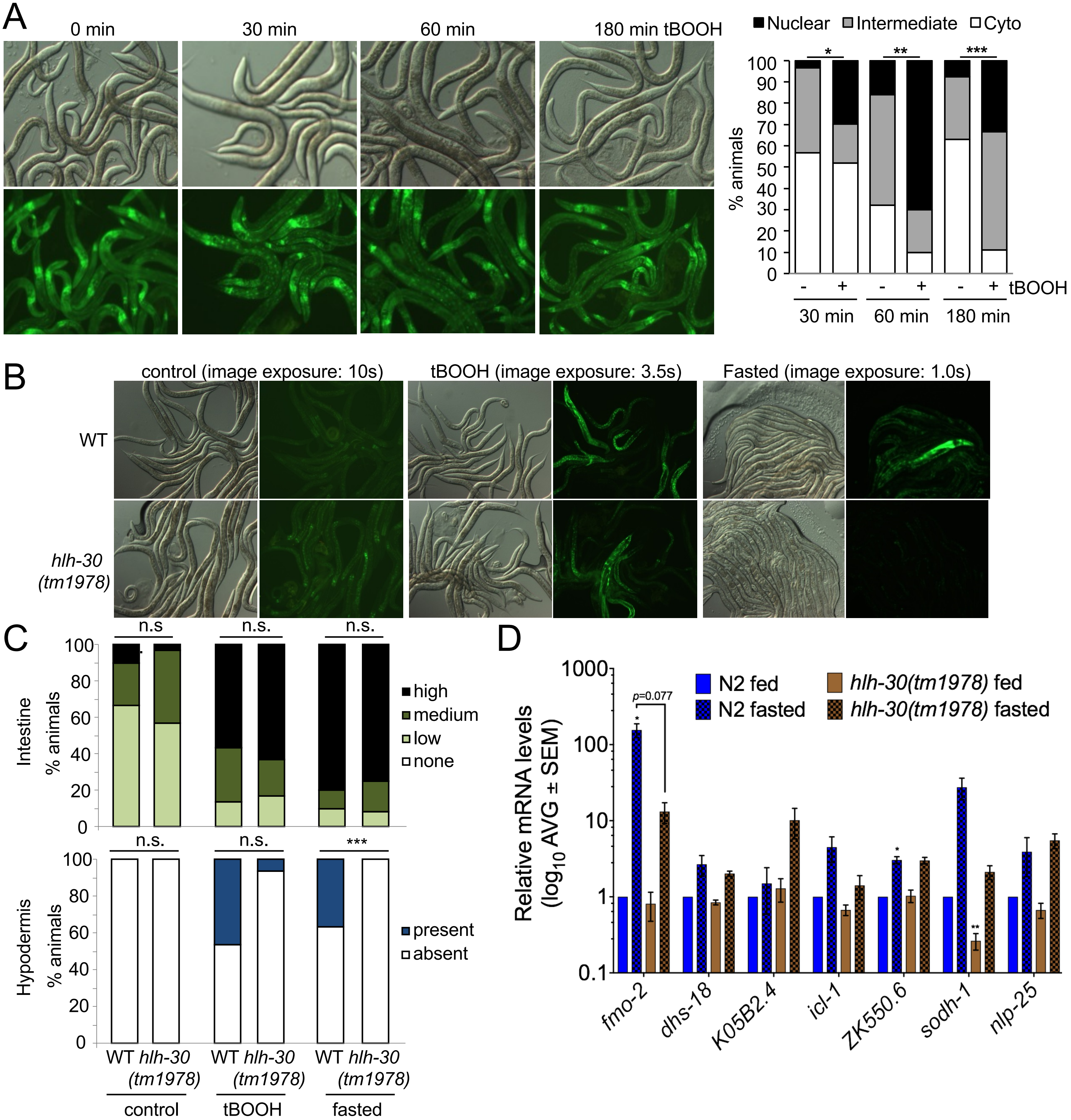

Fig. S3. Regulation of HLH-30 by stress, and requirements of *hlh-30* in stress-induced transcription.
[A] Left: HLH-30::GFP localization after exposure to 10mM tBOOH for indicated times. Right: Quantification; cyto = cytoplasmic. Statistically significant differences between groups of 25-30 tBOOH-exposed and control animals are indicated: *$p<0.05$, **$p<0.005$, and ***$p<0.0005$ (Chi-squared test).
[B] Imaging and **[C]** quantification of expression of *fmo-2p::gfp* in control, 10mM tBOOH (3h), and fasted (12h) *fmo-2p::gfp* and *hlh-30(tm1978); fmo-2p::gfp* worms. In [C], intestinal and hypodermal fluorescence of *fmo-2p::*GFP in wild-type and *hlh-30*(*tm1978*) backgrounds were quantified for groups of 24-30 animals and analyzed as in Figure S1B.
[D] Fold changes in mRNA levels (*vs.* untreated N2 wild-type) in L4 N2 and *hlh-30(tm1978)* worms fasted for 8 hours (n=3). *=$p<0.05$ and **=$p<0.01$, compared to wild-type fed worms, as determined by unpaired Student's t-test.

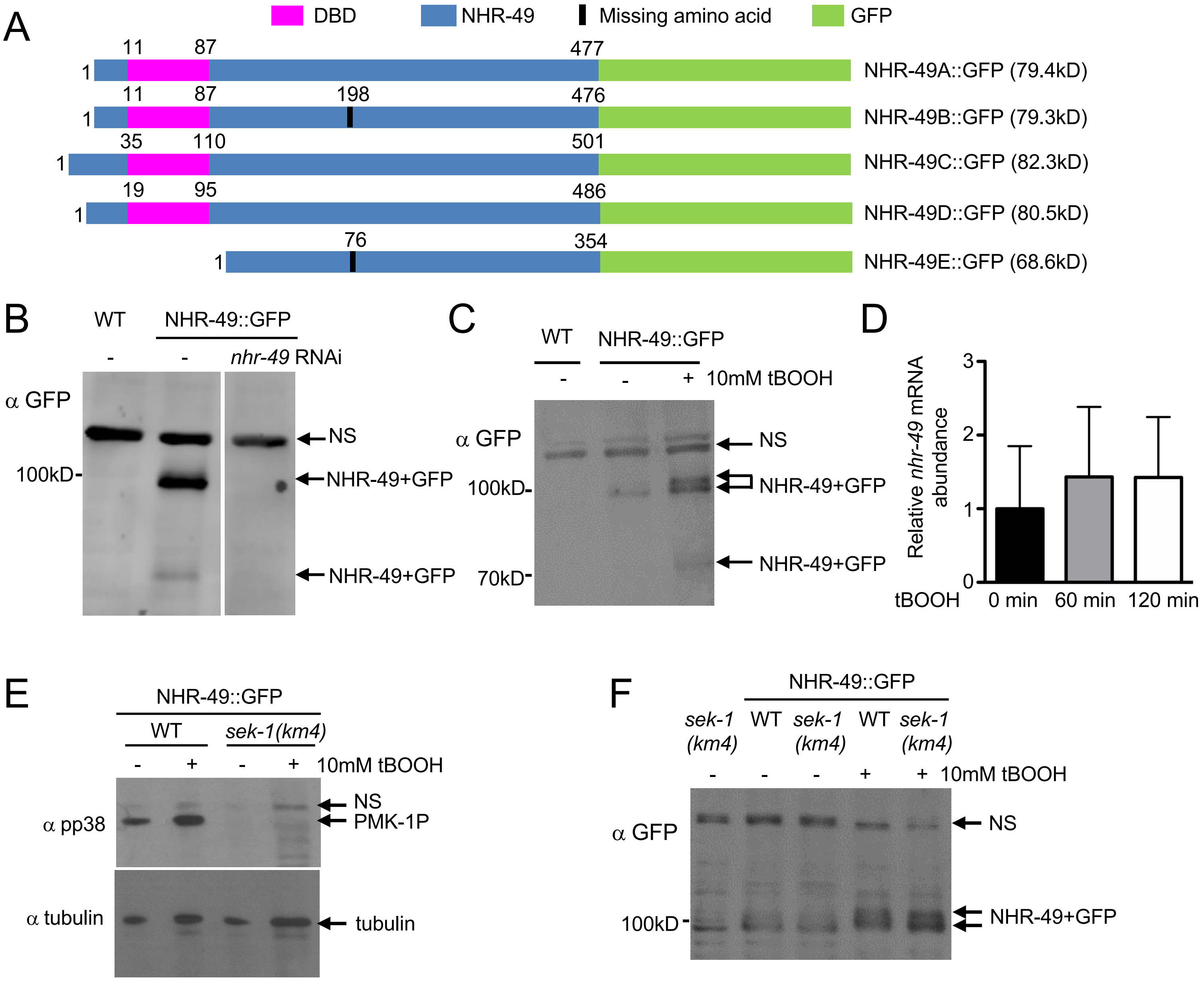

Fig. S4. NHR-49 isoforms and regulation of NHR-49 levels by tBOOH and *sek-1*.

[A] The *Pnhr-49::nhr-49::gfp* transgene is predicted to express all five NHR-49 isoforms (blue) as GFP fusions. The predicted MW of each isoform was calculated based on annotation in WormBase (WS258) plus the MW of GFP (27kD; green). The DNA binding domain (DBD; pink) is indicated; note, that NHR-49E encodes an isoform missing the DBD. ‘Missing amino acid’ indicates a residue not included in NHR-49B and NHR-49E.

[B-C] Anti-GFP immunoblot detects two NHR-49::GFP bands with apparent MWs of ~70 and ~100 kDa that are absent in non-transgenic worms [B], reduced by *nhr-49* RNAi [B], and induced by 1h treatment with 10mM tBOOH [C]. Non-specific bands (NS) show equal protein loading.

[D] Normalized *nhr-49* transcript abundance before and after exposure to 10mM tBOOH (n=3). Transcript levels are not significantly changed by exposure to tBOOH (determined by unpaired Student’s t-test).

[E] Western blot analysis of levels of phosphorylated PMK-1 (PMK-1P) in wild-type (WT) and *sek-1*(*km4)* mutant worms expressing the *Pnhr-49::nhr::gfp* extrachromosomal array before and after 10min exposure to 10mM tBOOH. Non-specific bands (NS) are indicated. Tubulin levels are indicated as a loading control.

[F] Western blot analysis of levels of NHR-49::GFP in wild-type (WT) and *sek-1*(*km4)* worms expressing the *Pnhr-49::nhr-49::gfp* extrachromosomal array before and after 1h exposure to 10mM tBOOH, as indicated. Non-specific bands (NS) indicate equal protein loading.

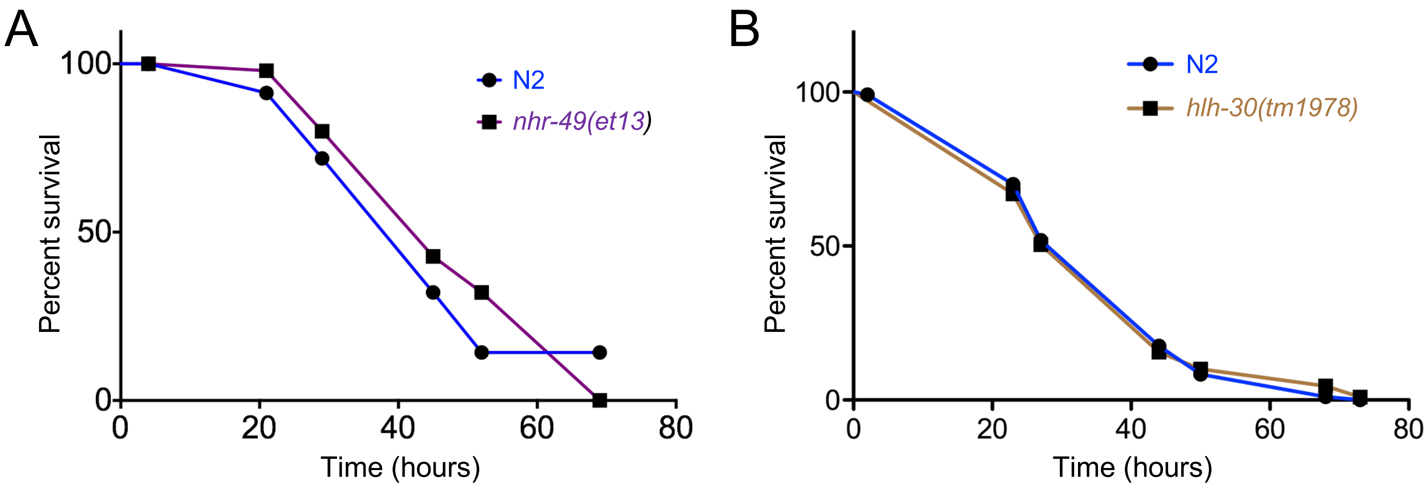

Fig. S5. Oxidative stress resistance phenotypes of *nhr-49(et13)* and *hlh-30(tm1978)* mutants.
[A] Survival plots of wild-type N2 and *nhr-49(et13)* worms on 6 mM tBOOH. Table S8 (Supporting information) shows statistics and replicates.
[B] Survival plots of wild-type N2 and *hlh-30(tm1978)* worms on 6 mM tBOOH. Table S10 (Supporting information) shows statistics and replicates.
