# Supplementary material for: NHR‐49/HNF4 integrates regulation of fatty acid metabolism with a protective transcriptional response to oxidative stress and fasting
Source: Aging Cell. 2018 Mar 5;17(3):e12743. doi: 10.1111/acel.12743 (PMC5946062; doi:10.1111/acel.12743)
Supplement: Supplementary file 2 [file ACEL-17-e12743-s002.pdf]

SUPPLEMENTAL TABLES

Table S1. List of transcription factor candidates that bind MDT-15 and were tested in the RNAi screen, and screen results.

**RNAi**	**Ref. for physical binding to MDT-15**	**Repeat 1**		**Repeat 2**		**Repeat 3**		**Verified using mounted images**		**Com-ments**
		Control	tBOOH	Control	tBOOH	Control	tBOOH	Control	tBOOH	
L4440		-	+	-	+	-	+	-	+	Neg. control
mdt-15		-	-	-	-	-	-	-	-	Pos. control
skn-1	(Goh et al. 2014)	-	++	-	++	-	+	-	++	
nhr-4	(Reece-Hoyes et al. 2013)	-	+	-	+	-	+			
nhr-8	(Arda et al. 2010)	-	+	-	+	-	+			
nhr-10	(Arda et al. 2010)	-	+	-	+	-	+			
nhr-12	(Arda et al. 2010)	+	+	+	+	-	+			
nhr-28	(Arda et al. 2010)					-	+			
nhr-49	(Taubert et al. 2006)	-	-	-	-	-	-	-	-	No fluores cence
nhr-64	(Taubert et al. 2006)	-	+	-	+	-	+			
nhr-69	(Arda et al. 2010)	-	+	-	+	-	+			
nhr-86	(Arda et al. 2010)	-	-	-	+	-	+			
nhr-97	ST unpubl.	-	+	-	+	-	+			
nhr-112	(Arda et al. 2010)					-	+			
nhr-114	(Arda et al. 2010)					+	+	+	+	↑ nucl. signal in -tBOOH
nhr-138	(Reece-Hoyes et al. 2013)					-	+			
nhr-273	(Arda et al. 2010)					-	+			
npax-2	(Arda et al. 2010)	-	+	-	+	-	+			
hlh-8	(Arda et al. 2010)	-	-	-	+	-	+			
ztf-2	(Arda et al. 2010)	-	+	-	+	-	+			
sbp-1	(Yang et al. 2006)			-	+	-	+	-	+	

Synchronized L1 stage worms were transferred to RNAi plates, allowed to grow to the young adult stage, and scored for fluorescence (control condition). 10-15 transgenic worms (assessed by transgenic Roller marker) were then transferred to

NGM-lite plates containing 10 mM tBOOH, exposed for 3 hours, and scored for fluorescence (tBOOH condition). Fluorescence was assessed using a Leica M205 FA fluorescence stereo microscope, and scored manually as low (-), high (+), or very high (++). For some RNAi clones, images were acquired using mounted worms and an inverted microscope.

Table S2. List of transcription factor candidates that bind the *fmo-2* promoter (according to modENCODE ChIP-seq data) and were tested in the RNAi screen, and screen results.

	**Control**		**10mM tBOOH (3 hrs)**		**Fasted (12 hrs)**	
**RNAi**	**Intestine**	**Hypodermis**	**Intestine**	**Hypodermis**	**Intestine**	**Hypodermis**
alr-1	-	-	-	-	-	-
mdl-1	↑↑	-	-	↓	-	↓
pqm-1	--	-	-	-	-	-
pha-4	↑↑↑	--	---	--	-	-
eor-1	↓-	-	-	↓	-	-
blmp-1	↑--	↓--	↓--	↓--	---	---
elt-3	↑-	--	--	↓-	↓-	↓-

In each experiment, 30 or more animals per treatment group were assessed; arrows and dashes indicate whether GFP signals were significantly increased (↑), decreased (↓), or unchanged (-) by treatment with the indicated RNAi compared negative control (L4440). Chi-squared distribution tests were used to determine whether differences between negative control (L4440) treated and transcription factor RNAi groups were statistically significant, i.e. $p = <0.05$.
