# Supplementary material for: NHR‐49/HNF4 integrates regulation of fatty acid metabolism with a protective transcriptional response to oxidative stress and fasting
Source: Aging Cell. 2018 Mar 5;17(3):e12743. doi: 10.1111/acel.12743 (PMC5946062; doi:10.1111/acel.12743)
Supplement: Supplementary file 5 [file ACEL-17-e12743-s005.pdf]

Table S5. Overlaps between genes induced by tBOOH, starvation, or in *glp-1(bn18)* mutants.

**Overlaps between gene sets**	250 tBOOH-induced genes (RNA-seq, this study,)
329 fasting-induced genes<br>(Uno et al. 2013)	Overlap: 44 genes<br>p-value < 2.2e-16<br>Odds ratio: 13.57
1306 genes induced in *glp-1(bn18)* mutants<br>(Steinbaugh et al. 2015)	Overlap: 61 genes<br>p-value < 2.2e-16<br>Odds ratio: 4.73
221 genes induced by tBOOH, SKN-1 independent<br>(Oliveira et al. 2009)	Overlap: 53 genes<br>p-value < 2.2e-16<br>Odds ratio: 29.14

Table S6: Oxidative stress (6 mM tBOOH) response of *C. elegans nhr-49* and *fmo-2* null mutants.

Strain	Experiment no.	Median survival (hours)	Number of subjects (Dx/Nx (Cx))	*p* value vs. wild type
N2	1	26	67/112 (45)	NA
	2	41	41/118 (77)	NA
	3	52	48/107 (59)	NA
	4	30	76/129 (53)	NA
nhr-49(nr2041)	1	20	79/92 (13)	0.0016**
	2	27.5	34/44 (10)	<0.001***
	3	Not determined		
	4	Not determined		
fmo-2(ok2147)	1	43	76/110 (34)	0.0015**
	2	51.5	32/66 (34)	0.0849
	3	69	36/113 (77)	0.0027**
	4	30	61/129 (68)	0.0363*

Number of subjects is denoted as follows: Dx=Number of deaths that occurred during the assay; Nx=Total number of animals used in assay; Cx=Number of censored events (i.e. worms that ruptured at the vulva, underwent internal hatching of the progeny, or crawled off the plate). All *p*-values are derived using the log-rank (Mantel-Cox) test. *p<0.05, **p<0.01, and ***p<0.001. NA=not applicable.

Table S7: Oxidative stress (6 mM tBOOH) response of *C. elegans Pnhr-49::nhr-49::gfp* worms and non-transgenic siblings.

Strain	Experiment no.	Median survival (hours)	Number of subjects (Dx/Nx (Cx))	*p* value vs. non-transgenic siblings
Non-transgenic siblings	1	45	87/106(19)	NA
	2	26	101/115(4)	NA
	3	46	92/107(15)	NA
	4	45	91/102(10)	NA
nhr-49p::nhr-49::gfp	1	52	66/104(32)	0.0015**
	2	44	89/116(3)	0.0015**
	3	28	90/110(18)	0.0653
	4	45	95/105(9)	0.0567

Number of subjects is denoted as follows: Dx=Number of deaths that occurred during the assay; Nx=Total number of animals used in assay; Cx=Number of censored events (i.e. worms that ruptured at the vulva, underwent internal hatching of the progeny, or crawled off the plate). All *p*-values are derived using the log-rank (Mantel-Cox) test. **p<0.01. NA=not applicable. Cox's regression analysis of all four datasets indicated that there was a statistically significant difference between *nhr-49p::nhr-49::gfp* and non-transgenic siblings: *p*=0.036.

Table S8: Oxidative stress (6 mM tBOOH) response of *C. elegans nhr-49(et13)* gof mutants.

Strain	Experiment no.	Median survival (hours)	Number of subjects (Dx/Nx (Cx))	*p* value vs. wild type

N2	1	45	81/118(37)	NA
	2	45	66/120(54)	NA
	3	47	66/116(50)	NA
	4	30	76/129 (53)	NA
nhr-49(et13)	1	45	54/119(65)	0.0492*
	2	52	39/136(97)	0.1352
	3	67	29/121(92)	0.0002***
	4	30	69/125 (56)	0.0007***

Number of subjects is denoted as follows: Dx=Number of deaths that occurred during the assay; Nx=Total number of animals used in assay; Cx=Number of censored events (i.e. worms that ruptured at the vulva, underwent internal hatching of the progeny, or crawled off the plate). All *p*-values are derived using the log-rank (Mantel-Cox) test. *p<0.05, and ***p<0.001. NA=not applicable. Cox's regression analysis of all four datasets indicated that there was a statistically significant difference between N2 and *nhr-49(et13)*: *p*<0.001.

Table S9: Oxidative stress (6 mM tBOOH) response of *C. elegans nhr-49*, *glp-1*, and *glp-1; nhr-49* mutants.

Strain	Experiment no.	Median survival (hours)	Number of subjects (Dx/Nx (Cx))	*p* value vs. wild type	*p* value vs. *glp-1(e2141)*
N2	1	23	57/116 (59)	NA	NA
	2	21	107/131 (24)	NA	NA
	3	30	65/90 (25)	NA	NA
	4	33	63/76 (13)	NA	NA
glp-1(e2141)	1	74	87/115 (28)	<0.0001****	NA
	2	69	84/104 (20)	<0.0001****	NA
	3	46	72/98 (26)	<0.0001****	NA
	4	46	85/113 (28)	<0.0001****	NA
nhr-49(nr2041)	1	Not determined			
	2	Not determined			
	3	23	60/76 (16)	<0.0001****	NA
	4	23	95/109 (14)	<0.0001****	NA
glp-1(e2141); nhr-49(nr2041)	1	30	62/63 (1)	0.0123*	<0.0001****
	2	27	64/66 (2)	<0.0001****	<0.0001****
	3	23	36/50 (14)	0.0101*	<0.0001****
	4	23	67/80 (13)	<0.0001****	<0.0001****

Number of subjects is denoted as follows: Dx=Number of deaths that occurred during the assay; Nx=Total number of animals used in assay; Cx=Number of censored events (i.e. worms that ruptured at the vulva, underwent internal hatching of the progeny, or crawled off the plate). All *p*-values are derived using the log-rank (Mantel-Cox) test. *p<0.05, and ****p<0.0001. NA=not applicable.

Table S10: Oxidative stress (6 mM tBOOH) response of *C. elegans* wild-type worms grown on RNAi clones targeting NHR-49 regulated genes.

RNAi clone	Experiment no.	Median survival (hours)	Number of subjects (Dx/Nx (Cx))	*p* value vs. control
control (L4440)	1	20	57/83(25)	NA
	2	24	54/81(32)	NA
	3	19	66/90(24)	NA
K05B2.4	1	20	67/74(7)	< 0.0001****
	2	20	70/84(7)	< 0.0001****
	3	19	62/81(19)	< 0.0001****
ZK550.6	1	28	49/71(21)	0.0036*
	2	20	60/81(30)	0.1735
	3	Undefined	17/74(55)	< 0.0001****
nlp-25	1	28	48/97(47)	< 0.0001****
	2	20	51/70(19)	0.049*
	3	19	67/83(13)	0.1737

dhs-18	1	28	44/100(55)	< 0.0001****
	2	20	54/93(35)	0.7438
	3	19	60/80(22)	0.7322
icl-1	1	28	37/102(63)	< 0.0001****
	2	20	60/88(24)	0.2671
	3	42	33/73(45)	< 0.0001****
sodh-1	1	20	59/81(19)	< 0.0001****
	2	20	61/70(8)	< 0.0001****
	3	19	57/72(15)	< 0.0001****

Number of subjects is denoted as follows: Dx=Number of deaths that occurred during the assay; Nx=Total number of animals used in assay; Cx=Number of censored events (i.e. worms that ruptured at the vulva, underwent internal hatching of the progeny, or crawled off the plate). All *p*-values are derived using the log-rank (Mantel-Cox) test. *p<0.05, and ****p<0.0001. NA=not applicable.

Table S11: Oxidative stress (6 mM tBOOH) response of *C. elegans glp-1* mutants grown on RNAi clones targeting NHR-49 regulated genes.

RNAi clone	Experiment no.	Median survival (hours)	Number of subjects (Dx/Nx (Cx))	*p* value vs. control
control (L4440)	1	74	61/97(34)	N/A
	2	55	39/70(29)	N/A
	3	42	94/111(22)	N/A
K05B2.4	1	28	81/104(13)	< 0.0001****
	2	47	78/102(26)	0.0125*
	3	19	75/103(27)	< 0.0001****
ZK550.6	1	47	58/74(18)	0.2017
	2	47	56/87(32)	0.9293
	3	42	50/63(13)	0.4061
nlp-25	1	74	66/99(30)	0.2711
	2	55	67/97(35)	0.1879
	3	42	83/95(16)	0.0669
dhs-18	1	47	84/103(21)	< 0.0001****
	2	74	73/88(22)	0.6203
	3	49	76/106(32)	0.0819
icl-1	1	74	52/99(50)	0.3797
	2	55	56/93(31)	0.9686
	3	27	68/103(34)	0.0052**
sodh-1	1	28	89/97(9)	< 0.0001****
	2	47	67/91(28)	< 0.0001****
	3	27	102/107(6)	< 0.0001****

Number of subjects is denoted as follows: Dx=Number of deaths that occurred during the assay; Nx=Total number of animals used in assay; Cx=Number of censored events (i.e. worms that ruptured at the vulva, underwent internal hatching of the progeny, or crawled off the plate). All *p*-values are derived using the log-rank (Mantel-Cox) test. *p<0.05, **p<0.01, and ****p<0.0001. NA=not applicable.

Table S12: Oxidative stress (6 mM tBOOH) response of *C. elegans hlh-30* mutants.

Strain	Experiment no.	Median survival (hours)	Number of subjects (Dx/Nx (Cx))	*p* value vs. wild type
N2	1	45	84/112(28)	NA
	2	46	83/94(2)	NA
	3	44	111/120(9)	NA
hlh-30(tm1978)	1	45	92/117(20)	0.8488
	2	46	88/94(2)	0.3601
	3	44	112/120(8)	0.8238

Number of subjects is denoted as follows: Dx=Number of deaths that occurred during the assay; Nx=Total number of animals used in assay; Cx=Number of censored events (i.e. worms that ruptured at the vulva, underwent internal hatching of the progeny, or crawled off the plate). All *p*-values are derived using the log-rank (Mantel-Cox) test. NA=not applicable.

Table S13. Worm strains used in this study.

Strain	Genotype	Reference
**N2**	Wild type	(Brenner 1974)
**STE68**	*nhr-49(nr2041) I*	(Van Gilst et al. 2005)
**VE40**	*N2 eavEx20[fmo-2p::gfp + rol-6(su1006)]*	This study
**STE108**	*nhr-49(et7) I*	(Lee et al. 2016)
**STE109**	*nhr-49(et8) I*	(Lee et al. 2016)
**STE110**	*nhr-49(et13) I*	(Lee et al. 2016)
**STE117**	*nhr-49(et13) I*; *eavEx20[fmo-2p::gfp + rol-6(su1006)]*	This study
**STE71**	*nhr-13(gk796) V*	(Pathare et al. 2012)
**STE69**	*nhr-66(ok940) IV*	(Pathare et al. 2012)
**STE70**	*nhr-80(tm1011) III*	(Goudeau et al. 2011)
**VC1668**	*fmo-2(ok2147) IV*	(Leiser et al. 2015)
**JIN1375**	*hlh-30(tm1978) IV*	(Lapierre et al. 2013)
**VE41**	*hlh-30(tm1978) IV; eavEx20[fmo-2p::gfp + rol-6(su1006)]*	This study
**MAH235**	*sqIs19[hlh-30p::hlh-30::gfp, rol-6(su1006)]*	(Lapierre et al. 2013)
**STE119**	*sqIs19[hlh-30p::hlh-30::gfp, rol-6(su1006)]; nhr-49(nr2041) I*	This study
**STE118**	*nhr-49(et13) I; hlh-30(tm1978) IV*	This study
**KAE9**	*seaSi39 [(pCFJ448) (eft-3p::fmo-2 + H2B::GFP) + Cbr-unc-119(+)] I; unc-119(ed3) III*	(Leiser et al. 2015)
**CF1903**	*glp-1(e2141ts) III*	(Arantes-Oliveira 2002)
**AGP22**	*nhr-49(nr2041) I; glp-1(e2141ts) III*	(Ratnappan et al. 2014)
**VE42**	*sek-1(km4); eavEx20[(fmo-2p::gfp + rol-6(su1006)]*	This study
**AGP24f**	*glmEx5 (Pnhr-49::nhr-49::GFP + Pmyo-2::mCherry)*	(Ratnappan et al. 2014)
**KU4**	*sek-1(km4) X*	(Tanaka-Hino et al. 2002)
**VE43**	*sek-1(km4) X; glmEx5[Pnhr-49::nhr-49::GFP + Pmyo-2::mCherry]*	This study
**KU25**	*pmk-1(km25) IV*	(Mizuno et al. 2004)

Table S14. List of non-Ahringer library RNAi clones used in this study.

Clone	Source
nhr-28	Custom made<br>Forward primer: GCTAGCgcggtgacaatatattccatgtt<br>Reverse primer: CCCGGGcCTCACTGCTTCGTTCGGAC
nhr-12	Vidal library, Plate 70 Well C03
nhr-86	(Arda et al. 2010)
nhr-112	Custom made<br>Forward primer: GCTAGCGAGGACAGGGATAGAgtagg<br>Reverse primer: AAGCTTGATATGTTTGAGTAGCctgg
nhr-114	Custom made<br>Forward primer: GCTAGCgttcagCTATCCGCTGTGCCTGC<br>Reverse primer: AAGCTTGAGTCCGGCTGGCctataacg
nhr-138	Custom made<br>Forward primer: GCTAGCGGCAACGAGGACAAAGgttag

Clone	Source
	Reverse primer: CCCGGGgttacCTGTACAGAAGTAAGC
nhr-273	Custom made<br>Forward primer: GCTAGCGCCGATTGATCAGAAGgtagg<br>Reverse primer: AAGCTTCGGACGGCGCATACAAGTTG

Table S15. List of qPCR primer sequences used in this study.

Gene	Forward primer (5'-3')	Reverse primer (5'-3')
fmo-2	GGAACAAGCGTGTTGCTGT	GCCATAGAGAAGACCATGTCG
dhs-18	CATCCAAAACTACCGGGAAC	TTTACTGCTGCCTCATCACG
K05B2.4	CCCTATACGAATGACAGGATTG	TGTTTGAACCTTGTGGTGAG
icl-1	ATTGCTTCGAGTTGATGAAGG	GATCCAAGCTGATCTTCGTAGTG
ZK550.6	TTCCGGAGCCAATAGAACTG	CGGGTCGAGACCATATCTTG
sodh-1	ATTGGTTGGAGGACACGAAG	GCTCGTGGCCTTTCTTACAG
nlp-25	ATCACTAATTGCGCTTCTCC	TCCTCCACCTCTGCCATAAC
nhr-49	TCCGAGTTCATTCTCGACG	GGATGAATTGCCAATGGAGC
hlh-30	CTCATCGGCCGGCGCTCATC	AGAACGCGATGCGTGGTGGG
mdt-15	GGAAATCCGTACAATCAGCAG	CAAGAACTGATGAAGGACCG
act-1	GCTGGACGTGATCTTACTGATTAC<br>C	GTAGCAGAGCTTCTCCTTGATGTC
tba-1	GTACACTCCACTGATCTCTGCTGA<br>CAAG	CTCTGTACAAGAGGCAAACAGCCAT<br>G
ubc-2	AGGGAGGTGTCTTCTTCCTCAC	CGGATTTGGATCACAGAGCAGC

SUPPLEMENTAL REFERENCES

Arantes-Oliveira N (2002) Regulation of Life-Span by Germ-Line Stem Cells in Caenorhabditis elegans. *Science* 295, 502–505.

Arda HE, Taubert S, Macneil LT, Conine CC, Tsuda B, Van Gilst M, Sequerra R, Doucette-Stamm L, Yamamoto KR & Walhout AJM (2010) Functional modularity of nuclear hormone receptors in a Caenorhabditis elegans metabolic gene regulatory network. *Molecular Systems Biology* 6, 367.

Brenner S (1974) The genetics of Caenorhabditis elegans. *Genetics* 77, 71–94.

Goh GYS, Martelli KL, Parhar KS, Kwong AWL, Wong MA, Mah A, Hou NS & Taubert S (2014) The conserved Mediator subunit MDT-15 is required for oxidative stress responses in Caenorhabditis elegans. *Aging Cell* 13, 70–79.

Goudeau J, Bellemin S, Toselli-Mollereau E, Shamalnasab M, Chen Y & Aguilaniu H (2011) Fatty Acid Desaturation Links Germ Cell Loss to Longevity Through NHR-80/HNF4 in C. elegans. *PLoS Biol* 9, e1000599.

Lapierre LR, De Magalhaes Filho CD, McQuary PR, Chu C-C, Visvikis O, Chang JT, Gelino S, Ong B, Davis AE, Irazoqui JE, Dillin A & Hansen M (2013) The TFEB orthologue HLH-30 regulates autophagy and modulates longevity in Caenorhabditis elegans. *Nat Commun* 4, 2267.

Lee K, Goh GYS, Wong MA, Klassen TL & Taubert S (2016) Gain-of-Function Alleles in Caenorhabditis elegans Nuclear Hormone Receptor nhr-49 Are Functionally Distinct. *PLoS ONE* 11, e0162708.

Leiser SF, Miller H, Rossner R, Fletcher M, Leonard A, Primitivo M, Rintala N, Ramos FJ, Miller DL & Kaeberlein M (2015) Cell nonautonomous activation of flavin-containing monooxygenase promotes longevity and health span.

Science 350, 1375–1378.

Mizuno T, Hisamoto N, Terada T, Kondo T, Adachi M, Nishida E, Kim DH, Ausubel FM & Matsumoto K (2004) The Caenorhabditis elegans MAPK phosphatase VHP-1 mediates a novel JNK-like signaling pathway in stress response. *EMBO J* 23, 2226–2234.

Oliveira RP, Abate JP, Dilks K, Landis J, Ashraf J, Murphy CT & Blackwell TK (2009) Condition-adapted stress and longevity gene regulation byCaenorhabditis elegansSKN-1/Nrf. *Aging Cell* 8, 524–541.

Pathare PP, Lin A, Bornfeldt KE, Taubert S & Van Gilst MR (2012) Coordinate Regulation of Lipid Metabolism by Novel Nuclear Receptor Partnerships K. Ashrafi, ed. *PLoS Genet* 8, e1002645.

Ratnappan R, Amrit FRG, Chen S-W, Gill H, Holden K, Ward J, Yamamoto KR, Olsen CP & Ghazi A (2014) Germline Signals Deploy NHR-49 to Modulate Fatty-Acid β-Oxidation and Desaturation in Somatic Tissues of C. elegans. *PLoS Genet* 10, e1004829.

Reece-Hoyes JS, Pons C, Diallo A, Mori A, Shrestha S, Kadreppa S, Nelson J, DiPrima S, Dricot A, Lajoie BR, Ribeiro PSM, Weirauch MT, Hill DE, Hughes TR, Myers CL & Walhout AJM (2013) Extensive rewiring and complex evolutionary dynamics in a C. elegans multiparameter transcription factor network. *Molecular Cell* 51, 116–127.

Steinbaugh MJ, Narasimhan SD, Robida-Stubbs S, Moronetti Mazzeo LE, Dreyfuss JM, Hourihan JM, Raghavan P, Operaña TN, Esmaillie R & Blackwell TK (2015) Lipid-mediated regulation of SKN-1/Nrf in response to germ cell absence. *elife* 4.

Tanaka-Hino M, Sagasti A, Hisamoto N, Kawasaki M, Nakano S, Ninomiya-Tsuji J, Bargmann CI & Matsumoto K (2002) SEK-1 MAPKK mediates Ca2+ signaling to determine neuronal asymmetric development in Caenorhabditis elegans. *EMBO Rep* 3, 56–62.

Taubert S, Van Gilst MR, Hansen M & Yamamoto KR (2006) A Mediator subunit, MDT-15, integrates regulation of fatty acid metabolism by NHR-49-dependent and -independent pathways in C. elegans. *Genes Dev* 20, 1137–1149.

Uno M, Honjoh S, Matsuda M, Hoshikawa H, Kishimoto S, Yamamoto T, Ebisuya M, Yamamoto T, Matsumoto K & Nishida E (2013) A fasting-responsive signaling pathway that extends life span in C. elegans. *Cell Rep* 3, 79–91.

Van Gilst MR, Hadjivassiliou H, Jolly A & Yamamoto KR (2005) Nuclear Hormone Receptor NHR-49 Controls Fat Consumption and Fatty Acid Composition in C. elegans. *PLoS Biol* 3, e53.

Yang F, Vought BW, Satterlee JS, Walker AK, Jim Sun Z-Y, Watts JL, DeBeaumont R, Saito RM, Hyberts SG, Yang S, Macol C, Iyer L, Tjian R, van den Heuvel S, Hart AC, Wagner G & Näär AM (2006) An ARC/Mediator subunit required for SREBP control of cholesterol and lipid homeostasis. *Nature* 442, 700–704.
